# X-ray source motion blur modeling and deblurring with generative diffusion for digital breast tomosynthesis

**DOI:** 10.1088/1361-6560/ad40f8

**Published:** 2024-05-14

**Authors:** Mingjie Gao, Jeffrey A Fessler, Heang-Ping Chan

**Affiliations:** 1 Department of Radiology, University of Michigan, Ann Arbor, MI 48109, United States of America; 2 Department of Electrical Engineering and Computer Science, University of Michigan, Ann Arbor, MI 48109, United States of America

**Keywords:** digital breast tomosynthesis, focal spot blur, image deblurring, image resolution, system modeling, x-ray source motion, diffusion models

## Abstract

*Objective*. Digital breast tomosynthesis (DBT) has significantly improved the diagnosis of breast cancer due to its high sensitivity and specificity in detecting breast lesions compared to two-dimensional mammography. However, one of the primary challenges in DBT is the image blur resulting from x-ray source motion, particularly in DBT systems with a source in continuous-motion mode. This motion-induced blur can degrade the spatial resolution of DBT images, potentially affecting the visibility of subtle lesions such as microcalcifications. *Approach*. We addressed this issue by deriving an analytical in-plane source blur kernel for DBT images based on imaging geometry and proposing a post-processing image deblurring method with a generative diffusion model as an image prior. *Main results*. We showed that the source blur could be approximated by a shift-invariant kernel over the DBT slice at a given height above the detector, and we validated the accuracy of our blur kernel modeling through simulation. We also demonstrated the ability of the diffusion model to generate realistic DBT images. The proposed deblurring method successfully enhanced spatial resolution when applied to DBT images reconstructed with detector blur and correlated noise modeling. *Significance*. Our study demonstrated the advantages of modeling the imaging system components such as source motion blur for improving DBT image quality.

## 1. Introduction

Digital breast tomosynthesis (DBT) has significantly improved the diagnosis of breast cancer due to its high sensitivity and specificity in detecting microcalcifications (MCs), masses and architectural distortions compared to two-dimensional (2D) mammography (Chan *et al* 2014, Chong *et al* 2019, Conant *et al* 2023). In DBT, the x-ray tube moves over a limited range of angles while acquiring a small number of low-dose projection views (PVs). The PVs are subsequently reconstructed into a quasi-three-dimensional (3D) tomographic image volume with an anisotropic voxel size such that the resolution is superior in the slices parallel to the detector but is very limited in the depth direction. However, one of the primary challenges in DBT imaging is the x-ray source motion blur that can degrade the quality of DBT images, reducing their sharpness and potentially affecting the visibility of subtle lesions such as MCs (Shaheen *et al* 2011, Marshall and Bosmans 2012, Zheng *et al* 2019, Lee and Baek 2022).

The x-ray tube motion in DBT can be carried out in two modes: step-and-shoot mode and continuous-motion mode (Sechopoulos 2013, Gao *et al* 2021b). In the step-and-shoot mode, the x-ray source essentially stops at each angle, acquires a PV, then moves to the next angle. The focal spot size of this mode equals the nominal size of a stationary source. Compared with the ideal point source, the nominal source has a negligible effect on the image sharpness (Zheng *et al* 2019). In continuous-motion mode, the x-ray source moves continuously along the designated arc while capturing PVs at the respective angles by pulsing the x-rays. This approach introduces source motion blur that depends on the pulse width, causing geometric unsharpness and substantially degrading the image resolution in the source motion direction (Marshall and Bosmans 2012, Zheng *et al* 2019). Very recently, Siemens introduced a flying focal spot technology for DBT to reduce the blurring effect of a continuously moving source (Michelfeit 2023). This new technology is outside the scope of our study.

X-ray source motion blur is not unique to DBT as a similar problem occurs in computed tomography (CT). Several image processing and reconstruction methods have been developed to address this issue. In CT, Tilley *et al* (2016) proposed to deconvolve the projection data for focal spot blur before reconstruction. In another study, Tilley *et al* (2018) modified the CT system forward model by incorporating focal spot blur as a shift-invariant convolution applied to the reconstructed images and used it in model-based iterative reconstruction (MBIR). Fu *et al* (2013) and Majee *et al* (2022) also modified the CT system forward model, but took a different approach by subsampling the focal spot and then averaging the projections. In DBT, Michielsen *et al* (2013) described the imaging process as projecting each DBT slice to the detector separately, convolving the slice projections with the slice-height-dependent source blur kernels, and then adding them up to obtain the final PV. They proposed to update the DBT slices sequentially for solving the reconstruction problem with their forward model. While their method increased the peak contrast-to-noise ratio of a simulated MC in a uniform background, the improvement was somewhat limited when applied to images with heterogeneous backgrounds.

Nonblind image deblurring is an important topic in image processing and computer vision research. Its task is to estimate sharp images from the blurred images given the blur kernel. Classic methods for nonblind image deblurring include the renowned Wiener filter (Wiener 1949) and Richardson-Lucy deconvolution (Richardson 1972). Model-based methods construct mathematical models and priors to estimate the latent sharp images using statistical methods like maximum *a posterior* (MAP). Great efforts have been devoted to designing image priors for MAP estimation (Krishnan and Fergus 2009, Zoran and Weiss 2011, Xu *et al* 2013). In recent years, deep convolutional neural network (CNN) methods have emerged as powerful tools, leveraging the capacity of deep networks to learn complex mappings from blurry to sharp images (Dong *et al* 2022, Zhang *et al* 2022). More recently, denoising diffusion probabilistic models (DDPM) and score-based generative models have gained significant attention for their ability to generate high-quality samples (Song and Ermon 2019, Ho *et al* 2020, Song *et al* 2021b). Their remarkable success in image generation facilitates various inverse problems including image deblurring (Kawar *et al* 2022, Saharia *et al* 2023, Wang *et al* 2023) and image reconstruction (Song *et al* 2022, Chung *et al* 2023).

X-ray source blur modeling for DBT remains a challenging problem due to its shift-variant nature. Furthermore, the low-dose exposure of DBT introduces a high level of image noise. In this study, we analytically derived the in-plane blur kernel for the reconstructed DBT slices using imaging geometry and showed that it could be approximated by a shift-invariant kernel for a given slice. We proposed an effective post-processing nonblind image deblurring approach with DDPM as an image prior and applied it to the reconstructed DBT images.

## 2. Methods

### 2.1. Source blur modeling

#### 2.1.1. DBT imaging system

**Figure 1.**
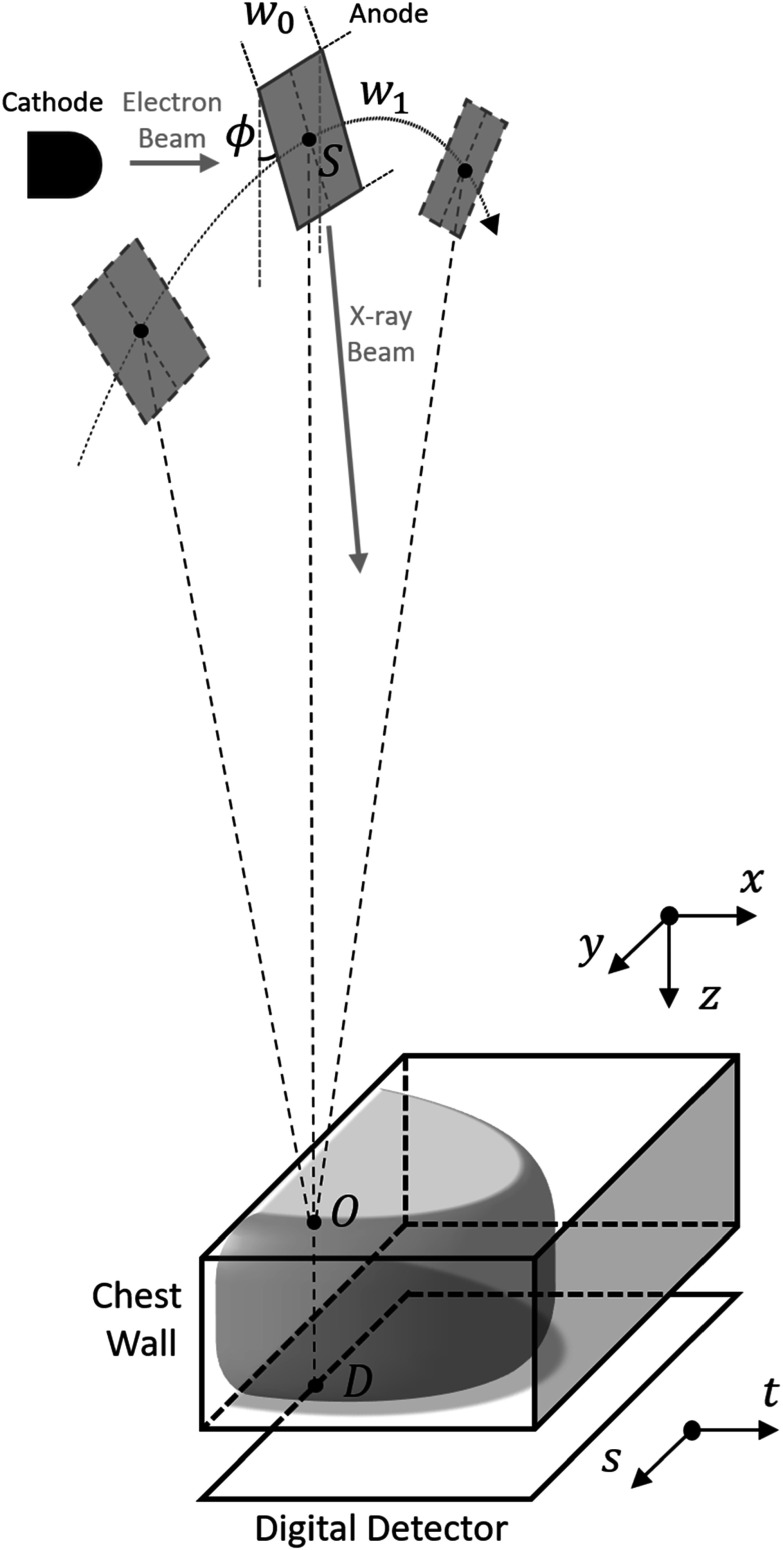
Diagram of DBT imaging system. The finite-sized rectangular x-ray source is exaggerated to show details.

Figure 1 shows a diagram of a DBT imaging system. We use $x$-$y$-$z$ coordinates for the imaged volume and $t$-$s$ coordinates for the digital detector. During the imaging process, the x-ray source moves around the compressed breast in the chest wall plane. The rotation center is denoted as $O.$ The source rotates over a limited angular range, and a total of ${N}_{p}$ PVs are captured. The raw projections available in clinical DBT systems usually have been preprocessed to correct for detector artifacts. We denote the source location for the central PV as $S$ and its vertical projection onto the detector plane as $D.$ Let ${\boldsymbol{v}}\in {{\mathbb{R}}}^{{N}_{x}{N}_{y}{N}_{z}}$ denote the (vectorized) DBT volume and ${{\boldsymbol{y}}}_{i}\in {{\mathbb{R}}}^{{N}_{t}{N}_{s}}$ denote the post-log PV at the $i$th scan angle. The forward imaging process can be characterized by the system matrices ${{\boldsymbol{A}}}_{i}\in {{\mathbb{R}}}^{{N}_{t}{N}_{s}\times {N}_{x}{N}_{y}{N}_{z}}$

\begin{eqnarray*}{{\boldsymbol{y}}}_{i}={{\boldsymbol{A}}}_{i}{\boldsymbol{v}}+{{\boldsymbol{n}}}_{i,y}\,i=1,\ldots ,{N}_{p},\end{eqnarray*}

where ${{\boldsymbol{n}}}_{i,y}{\sim }{\mathscr{N}}\left(0,{\sigma }_{i,y}^{2}{\boldsymbol{I}}\right)$ is the additive PV noise that follows the Gaussian distribution with a mean of 0 and standard deviation of ${\sigma }_{i,y}.$ We stack all the ${{\boldsymbol{A}}}_{i}$ matrices to define the overall system of equations

\begin{eqnarray*}\begin{array}{c}{\boldsymbol{y}}={\boldsymbol{Av}}+{{\boldsymbol{n}}}_{y}\,\mathrm{where}\,{\boldsymbol{A}}=\left[\begin{array}{c}{{\boldsymbol{A}}}_{1}\\ \vdots \\ {{\boldsymbol{A}}}_{{N}_{p}}\end{array}\right],\,{\boldsymbol{y}}=\left[\begin{array}{c}{{\boldsymbol{y}}}_{1}\\ \vdots \\ {{\boldsymbol{y}}}_{{N}_{p}}\end{array}\right],\,{{\boldsymbol{n}}}_{y}=\left[\begin{array}{c}{{\boldsymbol{n}}}_{1,y}\\ \vdots \\ {{\boldsymbol{n}}}_{{N}_{p},y}\end{array}\right].\end{array}\end{eqnarray*}

#### 2.1.2. In-plane source blur kernel estimation

The x-ray source is not a point source but has a finite size and shape. For simplicity, it is modeled as a rectangular source (edges ${w}_{0}$ and ${w}_{1},$ target angle $\phi $) with x-rays emitted uniformly across the anode target, as shown in figure 1. When the source rotates around $O,$ the edge ${w}_{0}$ stays parallel to the tangent line of the source trajectory. In x-ray imaging, a small $\phi $ is often used to keep the effective focal spot size small according to the line focus principle (Bushberg *et al* 2011). This study uses a nominal focal spot size of ${w}_{0}={w}_{1}\,\sin \phi =0.3\,{\mathrm{mm}},$ which is the nominal size for most mammography systems.

For the step-and-shoot mode, the source size is the same as the stationary nominal source size. However, for the continuous-motion mode, ${w}_{0}$ is elongated and equal to the nominal size convolved with the source traveling distance during the x-ray pulse. For example, the Siemens Mammomat Inspiration DBT system uses a continuous-motion x-ray source with a 0.18° motion angle per pulse for most exposures (Mackenzie *et al* 2017). Given that the distance between the source and rotation center is 600 mm, the effective source size ${w}_{0}$ is ${0.18}^{\circ }\times \left(\pi /{180}^{\circ }\right)\times 600\,{\mathrm{mm}}+0.3\,{\mathrm{mm}}=2.185\,{\mathrm{mm}}.$

**Figure 2.**
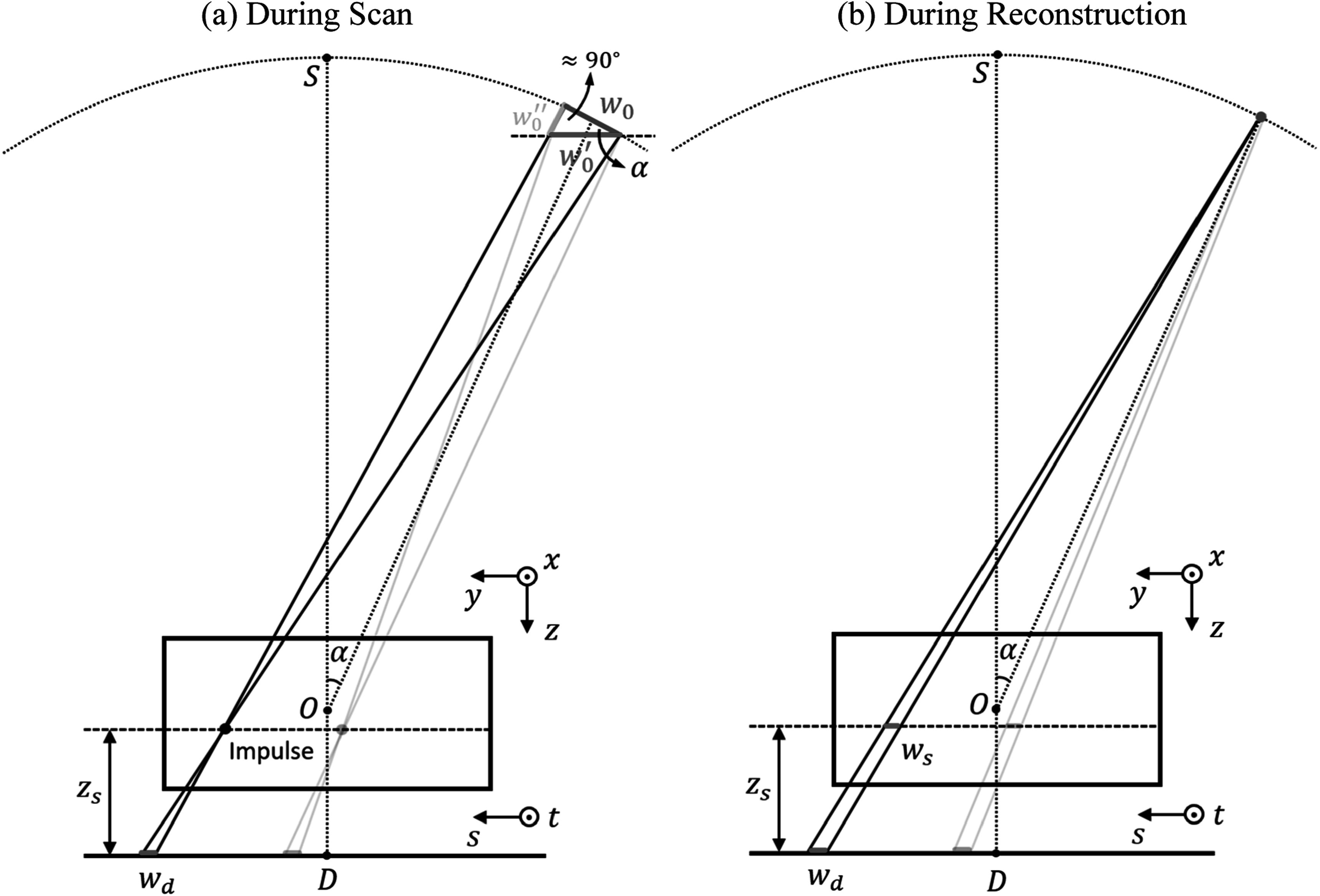
The simplification of source model and the derivation of in-plane source blur kernel. (a) For the simplified 1D line source ${w}_{0},$ we approximate it with ${w}_{0}^{{\prime} }$ and ignore the ${w}_{0}^{{\prime\prime} }$ component. Given an impulse at height ${z}_{s}$ above the detector, its PSF at the detector is ${w}_{d}.$ (b) In the reconstruction process, the in-plane PSF at the impulse location is ${w}_{s}$.

Zheng *et al* (2019) conducted a simulation study and demonstrated that a focal spot of the nominal size has negligible effect on the DBT image resolution compared with the ideal point source, while the extra blur caused by source motion leads to a substantial loss of image resolution in the source motion direction. Since the effective source dimension ${w}_{1}\sin \phi $ in the $x$-direction (the anode-cathode direction, i.e. the chestwall-anterior direction) remains at 0.3 mm at the chest wall regardless of the motion of x-ray source and decreases to less than 0.3 mm as the distance from the chest wall increases, the source blur in the $x$-direction has a negligible effect on image resolution (Zheng *et al* 2019). Therefore, the source model can be simplified by ignoring the $x$-dimension such that the rectangular source collapses to a line source. We focus on the continuous-motion x-ray source and consider a simplified 1D line source of length ${w}_{0}$ tangential to the source motion trajectory, as shown in figure 2(a).

During the scan process, at the scan angle $\alpha ,$ we consider an impulse with a distance ${z}_{s}$ from the detector (figure 2(a)). We assume that the impulse is far from the source, so the ray connecting the impulse and the end point of ${w}_{0}$ is perpendicular to ${w}_{0}.$ We further approximate ${w}_{0}$ with ${w}_{0}^{{\prime} }={w}_{0}/\cos \alpha $ that is parallel with the detector. Then, the point spread function (PSF) of the impulse at the detector is a line whose length ${w}_{d}\left({z}_{s},\alpha \right)$ can be obtained using triangle similarity

\begin{eqnarray*}{w}_{d}\left({z}_{s},\alpha \right)=\frac{{w}_{0}}{\cos \alpha }\cdot \frac{{z}_{s}}{{D}_{{SO}}\cdot \cos \alpha +{D}_{{OD}}-{z}_{s}},\end{eqnarray*}

where ${D}_{{SO}}$ is the distance between $S$ and $O,$ and ${D}_{{OD}}$ is the distance between $O$ and $D.$ Although the impulse is drawn to be on the source motion plane for simplicity, ${w}_{d}$ (the PSF of the impulse from ${w}_{0}^{{\prime} }$) is independent of the location of the impulse along both the $x$- and $y$-directions, i.e. shift-invariant, at a given ${z}_{s}.$ That is, the similar triangle relationship still holds if the impulse is moved to a different ${y}$ location, as depicted in gray in figure 2(a), or if the impulse is moved to a different $x$ location, ${w}_{d}$ remains parallel with ${w}_{0}^{{\prime} }$ and maintains the same ratio. Considering ${w}_{0}$ and ${w}_{0}^{{\prime} }$ as vectors in 3D when the impulse is moved to a general ($x,$ $y$) location in figure 2(a), the approximation of ${w}_{0}$ with ${w}_{0}^{{\prime} }$ results in the residual vector ${w}_{0}^{{\mathrm{{\prime} }}{\mathrm{{\prime} }}}$ that may cause a shift-variant blur when projected to the detector plane. However, this projected component is small and we assume it to be negligible in our modeling, as validated by our simulation study in section 4.1.

During reconstruction, the point source projector is normally used for forward and backward projections. Then, as shown in figure 2(b), the in-plane PSF of an impulse at the reconstructed DBT slice at ${z}_{s}$ is a line whose length ${w}_{s}\left({z}_{s},\alpha \right)$ is

\begin{eqnarray*}{w}_{s}\left({z}_{s},\alpha \right)={w}_{d}\left({z}_{s},\alpha \right)\cdot \frac{{D}_{{SO}}\cdot \cos \alpha +{D}_{{OD}}-{z}_{s}}{{D}_{{SO}}\cdot \cos \alpha +{D}_{{OD}}}=\frac{{w}_{0}}{\cos \alpha }\cdot \frac{{z}_{s}}{{D}_{{SO}}\cdot \cos \alpha +{D}_{{OD}}}.\end{eqnarray*}

Note that the PSF ${w}_{s}$ is also shift-invariant within the slice because ${w}_{d}$ is shift-invariant and the similar triangle relationship always holds no matter where the impulse is for a given ${z}_{s}.$

Equation (4) is the in-plane source blur PSF from one scan angle. The source motion blur in the reconstructed DBT images is a combined effect of the blurs from all scan angles. As shown in section 4.1, the lengths ${w}_{s}$ from different scan angles are close to their mean averaged over all angles

\begin{eqnarray*}{\mathop{w}\limits^{\unicode{x00305}}}_{s}\left({z}_{s}\right)=\frac{1}{{N}_{p}}{\sum }_{\alpha \in \left\{{\alpha }_{1},\ldots ,{\alpha }_{{N}_{p}}\right\}}{w}_{s}\left({z}_{s},\alpha \right).\end{eqnarray*}

Therefore, we treat the aggregated PSF over all scan angles also as a line with a length of ${\mathop{w}\limits^{\unicode{x00305}}}_{s}.$

To summarize, for a continuous-motion DBT system, the in-plane blur kernel caused by the source motion in the reconstructed DBT images is a line, the size of which is shift-invariant over a DBT plane at a given slice height. Mathematically, we define a block-diagonal matrix ${\boldsymbol{B}}$ to characterize the source motion blur and incorporate it into the DBT system of equation (2)

\begin{eqnarray*}\begin{array}{c}{\boldsymbol{y}}={\boldsymbol{ABv}}+{{\boldsymbol{n}}}_{y}\,\mathrm{where}\,{\boldsymbol{B}}=\left[\begin{array}{cccc}{{\boldsymbol{B}}}_{1} &amp; 0 &amp; \cdots &amp; 0\\ 0 &amp; {{\boldsymbol{B}}}_{2} &amp; \cdots &amp; 0\\ \vdots &amp; \vdots &amp; \ddots &amp; \vdots \\ 0 &amp; 0 &amp; \cdots &amp; {{\boldsymbol{B}}}_{{N}_{z}}\end{array}\right].\end{array}\end{eqnarray*}

The matrices ${{\boldsymbol{B}}}_{z}\in {{\mathbb{R}}}^{{N}_{x}{N}_{y}\times {N}_{x}{N}_{y}}$ represent the shift-invariant blur for each slice $z=1,\text{}\ldots ,\text{}{N}_{z}$ and can be efficiently implemented as convolution with kernel size ${\mathop{w}\limits^{\unicode{x00305}}}_{s}.$ Section 3.2 and section 4.1 present a simulation study to verify our derivation and justify our assumptions that simplify the DBT source blur model.

### 2.2. Nonblind image deblurring

Having introduced ${\boldsymbol{B}},$ a natural idea for addressing source motion blur is to replace ${\boldsymbol{A}}$ with the new system matrix ${\boldsymbol{AB}}$ in existing DBT reconstruction algorithms for forward and backward projections. However, the inherent limited-angle design of DBT makes the reconstruction inverse problem highly underdetermined. We found that this posed a substantial challenge, and using ${\boldsymbol{AB}}$ in DBT reconstruction did not improve image sharpness. Section 5 presents a simulation study to demonstrate the limitation of this approach.

An alternative approach is to separate ${\boldsymbol{B}}$ from ${\boldsymbol{A}}$ and turn the source blur modeling problem into post-processing deblurring. We formulate the deblurring problem as

\begin{eqnarray*}{{\boldsymbol{v}}}_{{\mathrm{blur}}}={\boldsymbol{B}}{{\boldsymbol{v}}}_{{\mathrm{true}}}+{{\boldsymbol{n}}}_{B},\end{eqnarray*}

where ${{\boldsymbol{v}}}_{{\mathrm{blur}}}$ denotes the reconstructed DBT images without any source blur modeling (i.e. by solving (2) instead of (6)), ${{\boldsymbol{v}}}_{{\mathrm{true}}}$ is the unknown sharp and clean images that we want to estimate, ${{\boldsymbol{n}}}_{B}{\sim }{\mathscr{N}}\left(0,{\sigma }_{B}^{2}{\boldsymbol{I}}\right)$ represents noise modeled as being additive Gaussian. This deblurring problem is nonblind because ${\boldsymbol{B}}$ is known.

When deblurring DBT images, it is crucial to control the image noise level through regularization due to the low-dose exposure of DBT scans. In this work, we investigated applying DDPM as an image prior for regularizing the deblurring process. The upcoming sections first give a brief review of DDPM, and then introduce the proposed deblurring method with generative diffusion.

#### 2.2.1. Denoising diffusion probabilistic models (DDPM)

DDPM is a class of generative models that use a diffusion process to model complex probability distributions (Sohl-Dickstein *et al* 2015, Ho *et al* 2020). These are Bayesian methods that assume that the images of interest can be represented as random vectors characterized by some probability distribution. In clinical practice, the radiologists view the DBT images as a series of in-focus planes parallel to the detector, so we consider the 2D slices ${{\boldsymbol{x}}}_{{\mathrm{true}}}\in {{\mathbb{R}}}^{{N}_{x}{N}_{y}}$ taken from ${{\boldsymbol{v}}}_{{\mathrm{true}}}$ as our images of interest with the associated distribution $p\left({{\boldsymbol{x}}}_{{\mathrm{true}}}\right).$ The diffusion process is a Markov chain that progressively adds noise to the image until a tractable distribution, such as a standard Gaussian, is achieved (Ho *et al* 2020). Mathematically, for a sequence of $T$ diffusion steps, at each step $t=1,\,\ldots ,\,T,$

\begin{eqnarray*}{{\boldsymbol{x}}}_{t}=\sqrt{1-{\beta }_{t}}{{\boldsymbol{x}}}_{t-1}+\sqrt{{\beta }_{t}}{\boldsymbol{\epsilon }},\end{eqnarray*}

where ${\beta }_{t}\in \left(\mathrm{0,1}\right)$ is the prescribed noise variance schedule, ${\boldsymbol{\epsilon }}{\sim }{\mathscr{N}}\left(0,{\boldsymbol{I}}\right)$ is the standard Gaussian noise. By the design of DDPM, we have ${{\boldsymbol{x}}}_{0}={{\boldsymbol{x}}}_{{\mathrm{true}}}$ and ${{\boldsymbol{x}}}_{T}{\sim }{\mathscr{N}}\left(0,{\boldsymbol{I}}\right).$ Equation (8) can also be written in a non-iterative form

\begin{eqnarray*}{{\boldsymbol{x}}}_{t}=\sqrt{{\bar{\alpha }}_{t}}{{\boldsymbol{x}}}_{0}+\sqrt{1-{\bar{\alpha }}_{t}}{\boldsymbol{\epsilon }},\end{eqnarray*}

where ${\mathop{\alpha }\limits^{\unicode{x00305}}}_{t}={\prod }_{{t}_{0}=1}^{t}\left(1-{\beta }_{{t}_{0}}\right).$

To reverse the diffusion process, DDPM trains a deep neural network ${{\boldsymbol{\epsilon }}}_{{\boldsymbol{\theta }}}\left({{\boldsymbol{x}}}_{t},t\right)$ parameterized by ${\boldsymbol{\theta }}$ to learn to predict the added noise from ${{\boldsymbol{x}}}_{t}.$ The training loss is a variant of a variational lower bound, and intuitively speaking, is the mean squared error between the predicted noise and actual added noise (Ho *et al* 2020)

\begin{eqnarray*}\mathop{\mathrm{argmin}}\limits_{{\boldsymbol{\theta }}}{{\mathbb{E}}}_{t,{{\boldsymbol{x}}}_{0},{\boldsymbol{\epsilon }}}\left[{\unicode{x02016}{\boldsymbol{\epsilon }}-{{\boldsymbol{\epsilon }}}_{{\boldsymbol{\theta }}}\left(\sqrt{{\bar{\alpha }}_{t}}{{\boldsymbol{x}}}_{0}+\sqrt{1-{\bar{\alpha }}_{t}}{\boldsymbol{\epsilon }},t\right)\unicode{x02016}}^{2}\right].\end{eqnarray*}

Once ${{\boldsymbol{\epsilon }}}_{{\boldsymbol{\theta }}}\left({{\boldsymbol{x}}}_{t},t\right)$ is trained, we can generate images by randomly initializing a sample with pure Gaussian noise ${{\boldsymbol{x}}}_{T}{\sim }{\mathscr{N}}\left(0,{\boldsymbol{I}}\right)$ and then iteratively removing noise from it following the DDPM sampling procedure (Ho *et al* 2020). In our implementation, we use a variant of DDPM sampling called denoising diffusion implicit models (DDIM) sampling (Song *et al* 2021a)

\begin{eqnarray*}{{\boldsymbol{x}}}_{t-1}=\sqrt{{\bar{\alpha }}_{t-1}}\left(\displaystyle \frac{{{\boldsymbol{x}}}_{t}-\sqrt{1-{\bar{\alpha }}_{t}}{{\boldsymbol{\epsilon }}}_{{\boldsymbol{\theta }}}\left({{\boldsymbol{x}}}_{t},t\right)}{\sqrt{{\bar{\alpha }}_{t}}}\right)+\sqrt{1-{\bar{\alpha }}_{t-1}}{{\boldsymbol{\epsilon }}}_{{\boldsymbol{\theta }}}\left({{\boldsymbol{x}}}_{t},t\right)\end{eqnarray*}

for $t=T,\ldots ,1.$

#### 2.2.2. Image deblurring with generative diffusion

The goal of image deblurring is to estimate the unknown sharp and clean images ${{\boldsymbol{x}}}_{{\mathrm{true}}}$ from the observed corrupted images ${{\boldsymbol{x}}}_{{\mathrm{blur}}}\in {{\mathbb{R}}}^{{N}_{x}{N}_{y}}$ taken from the DBT volume ${{\boldsymbol{v}}}_{{\mathrm{blur}}}.$ In Bayesian image deblurring, commonly used techniques include sampling from the posterior $p\left(\left.{{\boldsymbol{x}}}_{{\mathrm{true}}}\right|{{\boldsymbol{x}}}_{{\mathrm{blur}}}\right)$ and MAP estimation. Note that the prior $p\left({{\boldsymbol{x}}}_{{\mathrm{true}}}\right)$ is the distribution of true DBT slices, which can be effectively sampled by a well-trained DDPM using DBT images with no noise and blur.

We propose to perform posterior sampling to estimate ${{\boldsymbol{x}}}_{{\mathrm{true}}}$ from ${{\boldsymbol{x}}}_{{\mathrm{blur}}}.$ This requires us to modify the unconditional DDPM sampling to be a conditional sampling process. To do so, we exploit the connection between DDPM and score-based generative modeling following the derivation of Dhariwal and Nichol (2021). First, it has been shown that the DDPM network ${{\boldsymbol{\epsilon }}}_{{\boldsymbol{\theta }}}\left({{\boldsymbol{x}}}_{t},t\right)$ approximates the gradient of the log probability, also called the score function, of the distribution $p\left({{\boldsymbol{x}}}_{t}\right)$

\begin{eqnarray*}{{\mathrm{\nabla }}}_{{{\boldsymbol{x}}}_{t}}\mathrm{log}p\left({{\boldsymbol{x}}}_{t}\right)=-\frac{{{\boldsymbol{\epsilon }}}_{{\boldsymbol{\theta }}}\left({{\boldsymbol{x}}}_{t},t\right)}{\sqrt{1-{\mathop{\alpha }\limits^{\unicode{x00305}}}_{t}}}.\end{eqnarray*}

To see this, recall that $p\left({{\boldsymbol{x}}}_{t}{\mathrm{|}}{{\boldsymbol{x}}}_{0}\right)\sim {\mathscr{N}}\left(\sqrt{{\bar{\alpha }}_{t}}{{\boldsymbol{x}}}_{0},\left(1-{\bar{\alpha }}_{t}\right){\boldsymbol{I}}\right)$ from (9), so

\begin{eqnarray*}{\nabla }_{{{\boldsymbol{x}}}_{t}}\mathrm{log}p\left({{\boldsymbol{x}}}_{t}{\mathrm{|}}{{\boldsymbol{x}}}_{0}\right)=-\displaystyle \frac{{{\boldsymbol{x}}}_{t}-\sqrt{{\bar{\alpha }}_{t}}{{\boldsymbol{x}}}_{0}}{1-{\bar{\alpha }}_{t}}=-\displaystyle \frac{\left({{\boldsymbol{x}}}_{t}-\sqrt{{\bar{\alpha }}_{t}}{{\boldsymbol{x}}}_{0}\right)/\sqrt{1-{\bar{\alpha }}_{t}}}{\sqrt{1-{\bar{\alpha }}_{t}}}\approx -\displaystyle \frac{{{\boldsymbol{\epsilon }}}_{{\boldsymbol{\theta }}}\left({{\boldsymbol{x}}}_{t},t\right)}{\sqrt{1-{\bar{\alpha }}_{t}}}.\end{eqnarray*}

and thus, using the law of iterated expectation

\begin{eqnarray*}{{\mathrm{\nabla }}}_{{{\boldsymbol{x}}}_{t}}\mathrm{log}p\left({{\boldsymbol{x}}}_{t}\right)={{\mathbb{E}}}_{p\left({{\boldsymbol{x}}}_{0}\right)}\left[{{\mathrm{\nabla }}}_{{{\boldsymbol{x}}}_{t}}\mathrm{log}p\left({{\boldsymbol{x}}}_{t}{\mathrm{|}}{{\boldsymbol{x}}}_{0}\right)\right]\approx {{\mathbb{E}}}_{p\left({{\boldsymbol{x}}}_{0}\right)}\left[-\frac{{{\boldsymbol{\epsilon }}}_{{\boldsymbol{\theta }}}\left({{\boldsymbol{x}}}_{t},t\right)}{\sqrt{1-{\mathop{\alpha }\limits^{\unicode{x00305}}}_{t}}}\right]=-\frac{{{\boldsymbol{\epsilon }}}_{{\boldsymbol{\theta }}}\left({{\boldsymbol{x}}}_{t},t\right)}{\sqrt{1-{\mathop{\alpha }\limits^{\unicode{x00305}}}_{t}}},\end{eqnarray*}

which gives (12). Then, the score function of the conditional distribution $p\left({{\boldsymbol{x}}}_{t}|{{\boldsymbol{x}}}_{{\mathrm{blur}}}\right)$ becomes

\begin{eqnarray*}\begin{array}{ccc}{{\mathrm{\nabla }}}_{{{\boldsymbol{x}}}_{t}}\mathrm{log}p\left({{\boldsymbol{x}}}_{t}{\mathrm{|}}{{\boldsymbol{x}}}_{{\mathrm{blur}}}\right) &amp; = &amp; {{\mathrm{\nabla }}}_{{{\boldsymbol{x}}}_{t}}\mathrm{log}p\left({{\boldsymbol{x}}}_{t}\right)+{{\mathrm{\nabla }}}_{{{\boldsymbol{x}}}_{t}}\mathrm{log}p\left({{\boldsymbol{x}}}_{{\mathrm{blur}}}{\mathrm{|}}{{\boldsymbol{x}}}_{t}\right)\\ &amp; = &amp; -\frac{1}{\sqrt{1-{\mathop{\alpha }\limits^{\unicode{x00305}}}_{t}}}\mathop{\underbrace{\left({{\boldsymbol{\epsilon }}}_{{\boldsymbol{\theta }}}\left({{\boldsymbol{x}}}_{t},t\right)-\sqrt{1-{\mathop{\alpha }\limits^{\unicode{x00305}}}_{t}}\cdot {{\mathrm{\nabla }}}_{{{\boldsymbol{x}}}_{t}}\mathrm{log}p\left({{\boldsymbol{x}}}_{{\mathrm{blur}}}{\mathrm{|}}{{\boldsymbol{x}}}_{t}\right)\right)}}\limits_{\,{\widetilde{{\boldsymbol{\epsilon }}}}_{{\boldsymbol{\theta }}}\left({{\boldsymbol{x}}}_{t},t\right){\mathrm{:= }}\,}\end{array}\end{eqnarray*}

where Bayes rule gives $p\left({{\boldsymbol{x}}}_{t}|{{\boldsymbol{x}}}_{{\mathrm{blur}}}\right)=\frac{p\left({{\boldsymbol{x}}}_{t}\right){p}\left({{\boldsymbol{x}}}_{{\mathrm{blur}}}|{{\boldsymbol{x}}}_{t}\right)}{p\left({{\boldsymbol{x}}}_{{\mathrm{blur}}}\right)}$ and the gradient of $\mathrm{log}p\left({{\boldsymbol{x}}}_{{\mathrm{blur}}}\right)$ with respect to ${{\boldsymbol{x}}}_{t}$ vanishes because it does not depend on ${{\boldsymbol{x}}}_{t}.$

When $t$ is small, the structural details of ${{\boldsymbol{x}}}_{t}$ are close to ${{\boldsymbol{x}}}_{0},$ so we assume ${{\boldsymbol{x}}}_{{\mathrm{blur}}}\approx {\boldsymbol{B}}{{\boldsymbol{x}}}_{t}+{{\boldsymbol{n}}}_{B,t},$ ${{\boldsymbol{n}}}_{B,t}{\sim }{\mathscr{N}}\left(0,{\sigma }_{B,t}^{2}{\boldsymbol{I}}\right).$ We omit the subscript of ${\boldsymbol{B}}$ to simplify the notation, but it should be clear that ${\boldsymbol{B}}$ here is the blur matrix for a slice rather than a volume. The gradient of the data-fit term is therefore ${{\mathrm{\nabla }}}_{{{\boldsymbol{x}}}_{t}}\mathrm{log}p\left(\left.{{\boldsymbol{x}}}_{{\mathrm{blur}}}\right|{{\boldsymbol{x}}}_{t}\right)=-\frac{1}{{\sigma }_{B,t}^{2}}{{\boldsymbol{B}}}^{{\prime} }\left({\boldsymbol{B}}{{\boldsymbol{x}}}_{t}-{{\boldsymbol{x}}}_{{\mathrm{blur}}}\right)$ where $^{\prime} $ denotes matrix transpose. Finally, we insert this gradient into (13) and define the modified DDPM output

\begin{eqnarray*}{\widetilde{{\boldsymbol{\epsilon }}}}_{{\boldsymbol{\theta }}}\left({{\boldsymbol{x}}}_{t},t\right)={{\boldsymbol{\epsilon }}}_{{\boldsymbol{\theta }}}\left({{\boldsymbol{x}}}_{t},t\right)+\frac{\sqrt{1-{\mathop{\alpha }\limits^{\unicode{x00305}}}_{t}}}{{\sigma }_{B,t}^{2}}{{\boldsymbol{B}}}^{{\prime} }\left({\boldsymbol{B}}{{\boldsymbol{x}}}_{t}-{{\boldsymbol{x}}}_{{\mathrm{blur}}}\right).\end{eqnarray*}

We use this function in DDPM sampling to draw samples from the posterior $p\left(\left.{{\boldsymbol{x}}}_{{\mathrm{true}}}\right|{{\boldsymbol{x}}}_{{\mathrm{blur}}}\right)$ instead of $p\left({{\boldsymbol{x}}}_{{\mathrm{true}}}\right).$ The sampling equation (11) now becomes

\begin{eqnarray*}\begin{array}{l}{{\boldsymbol{x}}}_{t-1}\,=\,\sqrt{{\bar{\alpha }}_{t-1}}\left(\displaystyle \frac{{{\boldsymbol{x}}}_{t}-\sqrt{1-{\bar{\alpha }}_{t}}\,{\tilde{{\boldsymbol{\epsilon }}}}_{{\boldsymbol{\theta }}}}{\sqrt{{\bar{\alpha }}_{t}}}\right)+\sqrt{1-{\bar{\alpha }}_{t-1}}\,{\tilde{{\boldsymbol{\epsilon }}}}_{{\boldsymbol{\theta }}}\\ \,=\,\sqrt{{\bar{\alpha }}_{t-1}}\left(\displaystyle \frac{{{\boldsymbol{x}}}_{t}-\sqrt{1-{\bar{\alpha }}_{t}}\,{{\boldsymbol{\epsilon }}}_{{\boldsymbol{\theta }}}}{\sqrt{{\bar{\alpha }}_{t}}}\right)+\sqrt{1-{\bar{\alpha }}_{t-1}}\,{{\boldsymbol{\epsilon }}}_{{\boldsymbol{\theta }}}-\displaystyle \frac{\sqrt{1-{\bar{\alpha }}_{t}}}{{\sigma }_{B,t}^{2}}\left(\displaystyle \frac{\sqrt{1-{\bar{\alpha }}_{t}}}{\sqrt{1-{\beta }_{t}}}-\sqrt{1-{\bar{\alpha }}_{t-1}}\right)\cdot {\boldsymbol{B}}^{\prime} \left({\boldsymbol{B}}{{\boldsymbol{x}}}_{t}-{{\boldsymbol{x}}}_{\mathrm{blur}}\right)\\ \,\approx \,\sqrt{{\bar{\alpha }}_{t-1}}\left(\displaystyle \frac{{{\boldsymbol{x}}}_{t}-\sqrt{1-{\bar{\alpha }}_{t}}\,{{\boldsymbol{\epsilon }}}_{{\boldsymbol{\theta }}}}{\sqrt{{\bar{\alpha }}_{t}}}\right)+\sqrt{1-{\bar{\alpha }}_{t-1}}\,{{\boldsymbol{\epsilon }}}_{{\boldsymbol{\theta }}}-\lambda \cdot {\boldsymbol{B}}^{\prime} \left({\boldsymbol{B}}{{\boldsymbol{x}}}_{t}-{{\boldsymbol{x}}}_{\mathrm{blur}}\right)\end{array}\end{eqnarray*}

where we isolate ${{\boldsymbol{B}}}^{{\prime} }\left({\boldsymbol{B}}{{\boldsymbol{x}}}_{t}-{{\boldsymbol{x}}}_{{\mathrm{blur}}}\right)$ and replace its positive coefficient with a tuning parameter $\lambda $ to control the balance between noise reduction and data fidelity. We drop the $t$-dependency of $\lambda $ for easier tuning while still achieving empirically good results.

### 2.3. X-ray source motion deblurring for reconstructed DBTs

We have introduced a post-processing deblurring method for reconstructed DBT image slices through DDPM posterior sampling. We only run the sampling equation (15) for a small number of steps $t=\mathop{T}\limits^{\unicode{x00305}},\,\ldots ,\,1,$ where $\mathop{T}\limits^{\unicode{x00305}}\ll T$ to satisfy the small $t$ assumption. To deblur the entire DBT volume, we do slice-by-slice deblurring.

The proposed deblurring method is applicable to DBTs obtained from any reconstruction method. In this study, we investigate applying it to the model-based image reconstruction with detector blur and correlated noise modeling (DBCN) approach (Zheng *et al* 2018). By deblurring DBCN reconstructed images, the overall image reconstruction and post-processing pipeline represents a framework that employs both source motion blur and detector blur modeling.

## 3. Materials

### 3.1. DBT system configuration

We focus our study on the Siemens Mammomat Inspiration DBT system that takes 25 PVs from −25° to 25° scan angles in 2.08° intervals with ${D}_{{SO}}=600$ mm and ${D}_{{OD}}=50$ mm. The gap between the detector plane and the bottom of the compressed breast is 20 mm. The Siemens system uses a continuous-motion x-ray source with a typical 0.18° source motion angle (Mackenzie *et al* 2017), and we modeled it as a line source of length ${w}_{0}=2.185\,{\mathrm{mm}},$ as described in section 2.1.2. The detector pixel size is 0.085 mm × 0.085 mm.

### 3.2. Verification study of blur kernel modeling

**Figure 3.**
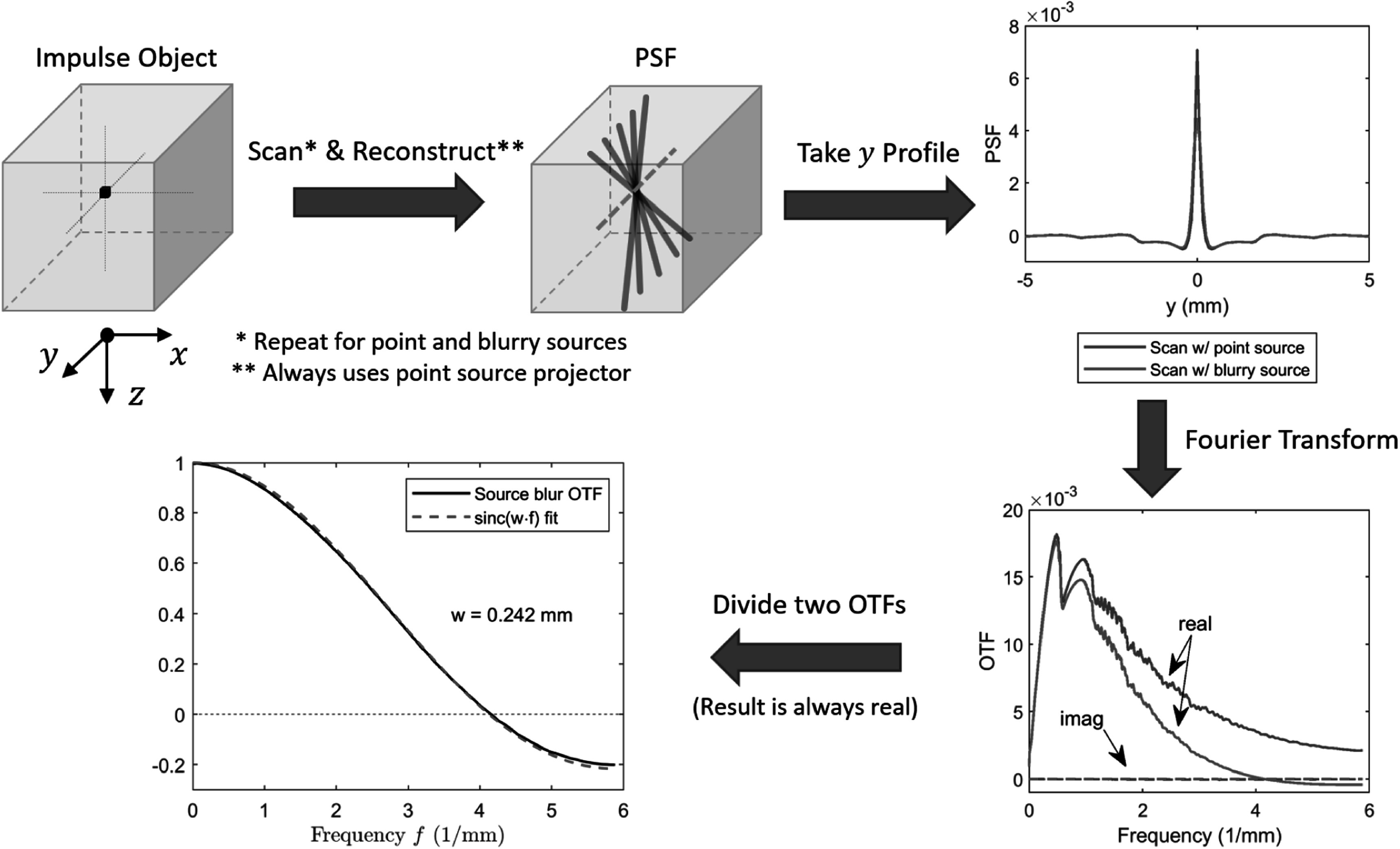
The simulation study to verify the in-plane blur kernel modeling. In this example, the impulse is centered in $y$-direction, so its PSF and OTF are both real and symmetric (only half of the OTF is shown in the plot). In general, if the impulse PSF is real but asymmetric, then the system OTF is conjugate symmetric and complex-valued. However, the source blur OTF is always real-valued because source blur does not introduce any phase change.

We designed a simulation study to verify the in-plane blur kernel modeling. Figure 3 shows the simulation workflow. First, we created an impulse object in the voxelized image with background value of zero. The impulse value was 0.05 mm^−1^, close to the typical attenuation coefficient of breast tissues (Johns and Yaffe 1987). Then, we simulated the PVs using the point and blurry sources, denoted as ${{\boldsymbol{y}}}_{{\mathrm{pt}}}$ and ${{\boldsymbol{y}}}_{{\mathrm{blur}}},$ respectively. The pixel values of ${{\boldsymbol{y}}}_{{\mathrm{pt}}}$ were generated with the segmented separable footprint (SG) projector (Zheng *et al* 2017) instead of simple ray-tracing. The generation of ${{\boldsymbol{y}}}_{{\mathrm{blur}}}$ used 50 SG projectors as sub-focal spots within ${w}_{0}$ uniformly. Because the blur occurs after the x-ray is attenuated in actual scans, we summed the sub-PVs in the pre-log domain to match the physics process and then took log to get the post-log blurry PVs. The simulation was noise-free.

Next, we reconstructed the PSFs with a point source SG projector for the following two conditions: $\mathop{{\mathrm{argmin}}}\limits_{{\boldsymbol{v}}}\frac{1}{2}{\unicode{x02016}{\boldsymbol{Av}}-{{\boldsymbol{y}}}_{{\mathrm{pt}}}\unicode{x02016}}^{2}$ and $\mathop{{\mathrm{argmin}}}\limits_{{\boldsymbol{v}}}\frac{1}{2}{\unicode{x02016}{\boldsymbol{Av}}-{{\boldsymbol{y}}}_{{\mathrm{blur}}}\unicode{x02016}}^{2}.$ The reconstructed PSFs had spoke-like inter-plane artifact due to the limited-angle nature of DBT. We took the 1D PSFs through the impulse in the $y$-direction and took Fourier transform to obtain the impulse optical transfer functions (OTFs). Although the OTFs are complex-valued in general because the PSFs are asymmetric (except when the impulse is centered in $y$), their ratio, which represents the source blur OTF, is always real-valued.

Finally, recall that the Fourier transform of a 1D rectangle function $\frac{1}{w}{\mathrm{rect}}\left(\frac{u}{w}\right)$ of width $w$ and unit area is a sinc function ${\mathrm{sinc}}\left(w\cdot f\right),$ where $u$ and $f$ are function variables. We fit the source blur OTF with ${\mathrm{sinc}}\left(w\cdot f\right)$ to estimate the blur kernel length ${w}$ and compared it with our analytically derived blur kernel length ${\mathop{w}\limits^{\unicode{x00305}}}_{s}$ defined in (5). We moved the impulse object to different $x,$ $y,$ or $z$ locations in the volume and repeated the experiment.

### 3.3. Data sets

#### 3.3.1. VICTRE phantoms

We used the Virtual Imaging Clinical Trial for Regulatory Evaluation (VICTRE) package (Badano *et al* 2018) to create virtual phantoms with breast tissue backgrounds to train the DDPM network and test the deblurring methods. The VICTRE package can generate anthropomorphic breast phantoms that were used for virtual clinical trials. The VICTRE virtual phantoms were defined on a 3D fine grid where each voxel was assigned with a label indicating its material. The voxel size was 0.05 mm × 0.05 mm × 0.05 mm. The PVs of the virtual phantoms were simulated by MC-GPU, a Monte Carlo x-ray imaging simulator in the package. MC-GPU was configured for the Siemens Mammomat Inspiration DBT system, and its simulation accuracy had been validated in terms of noise and resolution (Badal *et al* 2021). It also provided an option of either using an ideal point source or a blurry source with 0.3 mm nominal size and 0.18° motion angle for the scans. The x-ray exposure was adaptively determined for each phantom by first running a quick scan with a small exposure, and then the full scan with a scaled exposure so that the mean glandular dose matched that of a real scan under automatic exposure control (AEC) (PHE 2018). MC-GPU assumed constant tube current for each PV. Scatter was simulated by MC-GPU, but we did not correct for scatter in this study.

For DDPM training, we created 70 virtual phantoms whose density and size characteristics are shown in table 1. The glandular volume fraction (GVF) setting followed that of the VICTRE study (Badano *et al* 2018). According to the formulation of the deblurring problem (7), ${{\boldsymbol{x}}}_{{\mathrm{true}}}$ (or ${{\boldsymbol{v}}}_{{\mathrm{true}}}$) represented the sharp and noiseless images. Hence, we used the point source in MC-GPU, and increased the exposure to be 5 times the AEC to better represent the ‘noiseless’ prior DBT image distribution $p\left({{\boldsymbol{x}}}_{{\mathrm{true}}}\right).$ We reconstructed the DBTs using DBCN (5 iterations, regularization strength = 70) and the SG projector (Zheng *et al* 2018). The reconstructed image voxel size was 0.085 mm × 0.085 mm × 1.0 mm. Due to the large sizes of DBT images, we trained the DDPM network using image patches instead of full DBT slices to reduce memory cost. We previously investigated the patch sizes of 32 × 32 pixels, 64 × 64 pixels, 128 × 128 pixels, and 256 × 256 pixels. All the patch sizes were found to work well for training; the network trained with 64 × 64-pixel patches generated more realistic looking images with a reasonable amount of training time compared to other patch sizes. We randomly extracted 128,401 64 × 64-pixel non-overlapping 2D slice patches from the reconstructed DBT images to form the DDPM training set.

**Table 1.** Density and size characteristics of the virtual breast phantoms for DDPM training.

Density	Almost entirely fatty	Scattered fibroglandular dense	Heterogeneously dense	Extremely dense
GVF	5%	15%	34%	60%
No. of phantoms	10	10	25	25
Thickness after compression (mm)	52–70 (2 mm intervals)	46–64 (2 mm intervals)	36–60 (1 mm intervals)	31–55 (1 mm intervals)

To test the deblurring methods, we created a separate set of virtual test phantoms. We considered two breast sizes: an average breast, and a large breast that maximized the source blur effect. The average breast had a diameter of 105 mm at the chest wall before compression (with a cone-like shape) and a thickness of 60 mm after compression. The large breast had a diameter of 120 mm before compression and a thickness of 80 mm after compression. We created 4 phantoms for both sizes, one at each breast density, resulting in a total of 8 test phantoms. The phantoms were embedded with a line pair test object discussed next. We scanned the phantoms twice under AEC exposure in MC-GPU, first using the blurry source, and then using the ideal point source to serve as a reference standard. The DBT images of the test phantoms were also reconstructed using DBCN (5 iterations, regularization strength = 70) and the SG projector.

#### 3.3.2. Line pair test object and image quality metrics

**Figure 4.**
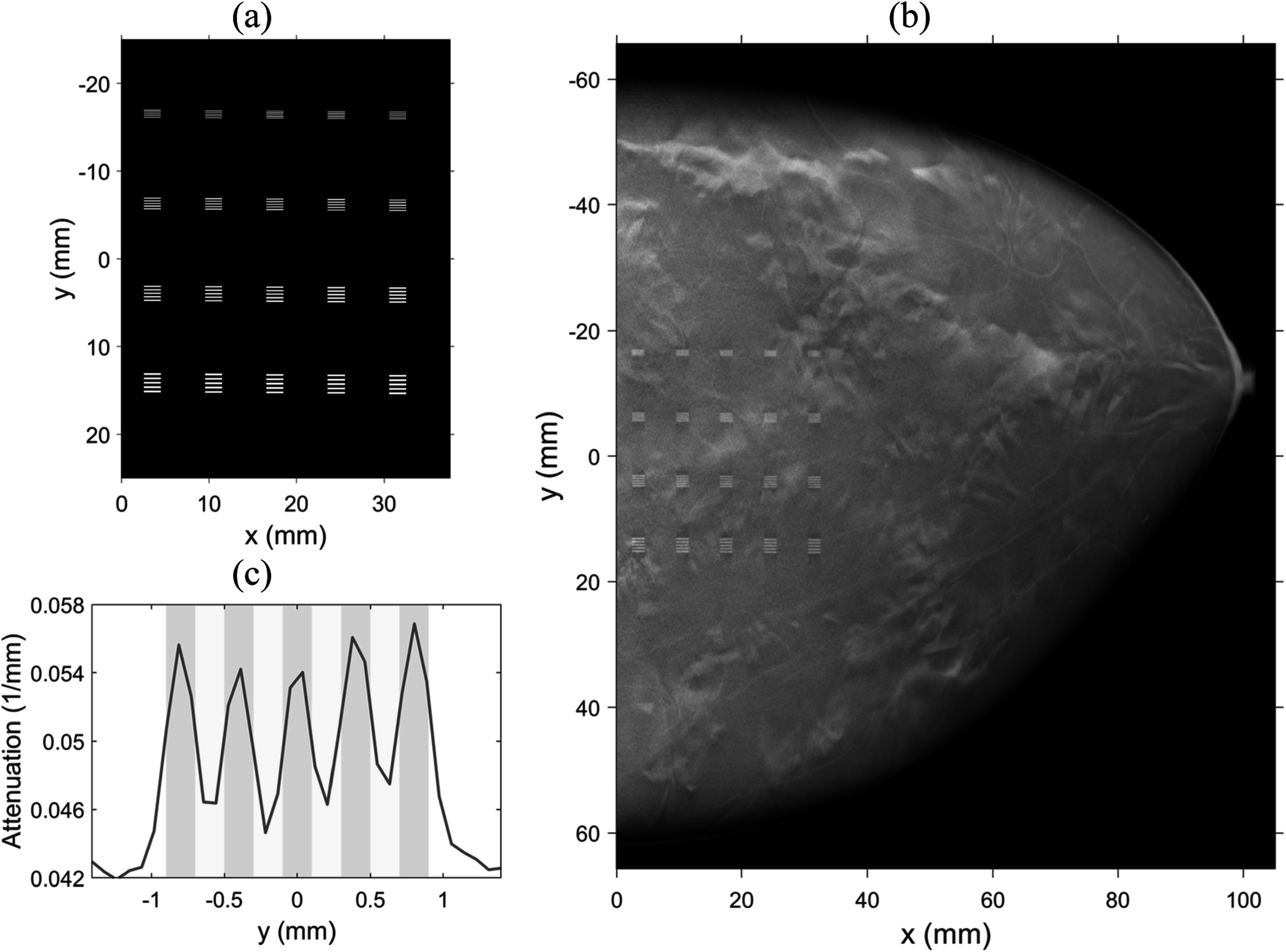
(a) LP test object with 5 LP/mm, 3.33 LP/mm, 2.5 LP/mm, and 2 LP/mm (top to bottom) and vertical shifts (left to right). (b) Example reconstructed DBT slice with embedded LP test object. (c) Illustration of LP contrast calculation (gray area: bar regions; yellow area: space regions).

To quantitatively evaluate the image resolution, we designed a test object consisting of line pairs (LPs) with a range of spatial frequencies, as shown in figure 4(a). The test object was 35 mm × 35 mm × 0.2 mm in size and voxelized with size of 0.05 mm × 0.05 mm × 0.05 mm (same as the virtual phantoms). The LPs were 0.2 mm in thickness and 2 mm in length and were made of calcium oxalate to mimic the attenuation of small MCs. Each LP group contained five horizontally placed bars with equal width and spacing. From top to bottom of the test object, the four bar width settings were 0.1 mm, 0.15 mm, 0.2 mm, and 0.25 mm. These were equivalent to the spatial frequencies of 5 LP/mm, 3.33 LP/mm, 2.5 LP/mm, and 2 LP/mm, respectively. The line pair groups were placed 10 mm apart in the vertical directions. Since the alignment between the test object and reconstruction voxel grid could affect the resolution of the reconstructed LPs, we duplicated the LPs in five columns from left to right of the test object with an accumulation of 0.05 mm vertical shift, simulating the possible random alignments of small object with the detector pixel array during imaging. The columns of the line pair groups were 7 mm apart in the horizontal direction.

To insert the LP test object into the virtual test phantoms, we assigned the corresponding phantom voxels with the label of calcium oxalate. The test object was parallel to the detector at a specified height from the detector and the bars were perpendicular to the source motion direction. The test object was close to the chest wall and centered in the $y$-direction, and was inserted well within the breast. Then, we simulated the PVs and reconstructed the images, as described in section 3.3.1. Figure 4(b) shows an example of a reconstructed DBT slice containing the test object.

We calculated the LP contrast and image noise of the reconstructed DBT images as image quality metrics. For each reconstructed LP, we averaged the central 1.5 mm bar region along $x$-direction to get the LP profile in $y$-direction. Then, we overlapped the LP profile with the ground truth locations of the five bars and four spaces on the continuous $y$ coordinate, as illustrated in figure 4(c). The profile of the reconstructed LP was linearly interpolated (lines connecting adjacent pixel values). If the reconstructed LP was well-resolved, it should have peaks and valleys matching the corresponding locations in the ideal profile. We followed Zheng *et al* (2019) and defined the contrast of the reconstructed LP as the difference between the mean value of the five ground truth bar regions (gray area in figure 4(c)) and the mean value of the four space regions (yellow area in figure 4(c)), normalized to the contrast of the input ideal profile. Since the input ideal profile had the same contrast for all LP frequencies, the relative contrast of the LP frequencies would be the same with or without normalization. The final contrast of each LP frequency was averaged over the five shifted instances in the test object. To quantify image noise, we took 20 10 × 10-pixel LP-free regions of interest (ROIs) near the LPs and calculated the root-mean-square pixel variation of the ROIs after background removal using quadratic fitting (Gao *et al* 2023). The overall image noise level was the average over all noise ROIs.

### 3.4. DDPM implementation

The structure of the DDPM network was a modified U-Net (Ronneberger *et al* 2015) as described in Ho *et al* (2020). The U-Net had four downsampling scales, each with three ResNet blocks (He *et al* 2016). The numbers of 3 × 3 convolutional kernels for the downsampling scales were 64, 128, 128, 128, respectively. Following Ho *et al* (2020), we trained the DDPM using 60 training epochs, a batch size of 256, and a learning rate of ${10}^{-4}$ with Adam optimizer (Kingma and Ba 2015). The noise variance schedule ${\beta }_{t}$ was evenly spaced between ${\beta }_{0}={10}^{-4}$ and ${\beta }_{T}=0.02$ with $T$ = 1000 (Ho *et al* 2020). The diffusion steps $t$ were encoded with sinusoidal positional encoding (Vaswani *et al* 2017) and then added to the feature maps of the ResNet blocks. We removed all the attention layers, so the network contained only convolution and up/downsampling layers, and thus could process images of arbitrary sizes. Section 4.3.1 discusses the parameter selection of $\mathop{T}\limits^{\unicode{x00305}}$ and $\lambda $ in the DDPM posterior sampling for deblurring.

### 3.5. Comparison methods

We compared the proposed deblurring method with the following nonblind deblurring methods: Tikhonov regularized deblurring (Tikhonov 1963, Gunturk and Li 2013), total variation (TV) regularized deblurring, and the unfolding super-resolution network (USRNet) (Zhang *et al* 2020). Tikhonov regularized deblurring was formulated as $\hat{{\boldsymbol{v}}}=\mathop{{\mathrm{argmin}}}\limits_{{\boldsymbol{v}}}\frac{1}{2}{\unicode{x02016}{\boldsymbol{Bv}}-{{\boldsymbol{v}}}_{{\mathrm{blur}}}\unicode{x02016}}^{2}+\frac{{\mu }_{{\mathrm{Tik}}}}{2}{\unicode{x02016}{\boldsymbol{v}}-{{\boldsymbol{v}}}_{{\mathrm{blur}}}\unicode{x02016}}^{2},$ which has an analytical solution that uses an inverse filter: $\hat{{\boldsymbol{v}}}={\left({{\boldsymbol{B}}}^{{\prime} }{\boldsymbol{B}}+{\mu }_{{\mathrm{Tik}}}{\boldsymbol{I}}\right)}^{-1}\left({{\boldsymbol{B}}}^{{\prime} }+{\mu }_{{\mathrm{Tik}}}{\boldsymbol{I}}\right){{\boldsymbol{v}}}_{{\mathrm{blur}}}.$ TV regularized deblurring was formulated as $\hat{{\boldsymbol{v}}}=\mathop{{\mathrm{argmin}}}\limits_{{\boldsymbol{v}}}\frac{1}{2}{\unicode{x02016}{\boldsymbol{Bv}}-{{\boldsymbol{v}}}_{{\mathrm{blur}}}\unicode{x02016}}^{2}+{\mu }_{{\mathrm{TV}}}{\unicode{x02016}{\boldsymbol{Dv}}\unicode{x02016}}_{1}$ where ${\boldsymbol{D}}$ contains the finite difference operators in $x$- and $y$- directions. USRNet was an end-to-end trainable CNN, and we trained it using the paired virtual phantom images with low-quality images being the simulated 1 × AEC exposure using a blurry source and high-quality images being the simulated 5 × AEC exposure using a point source.

## 4. Results

### 4.1. Verification study of blur kernel modeling

**Figure 5.**
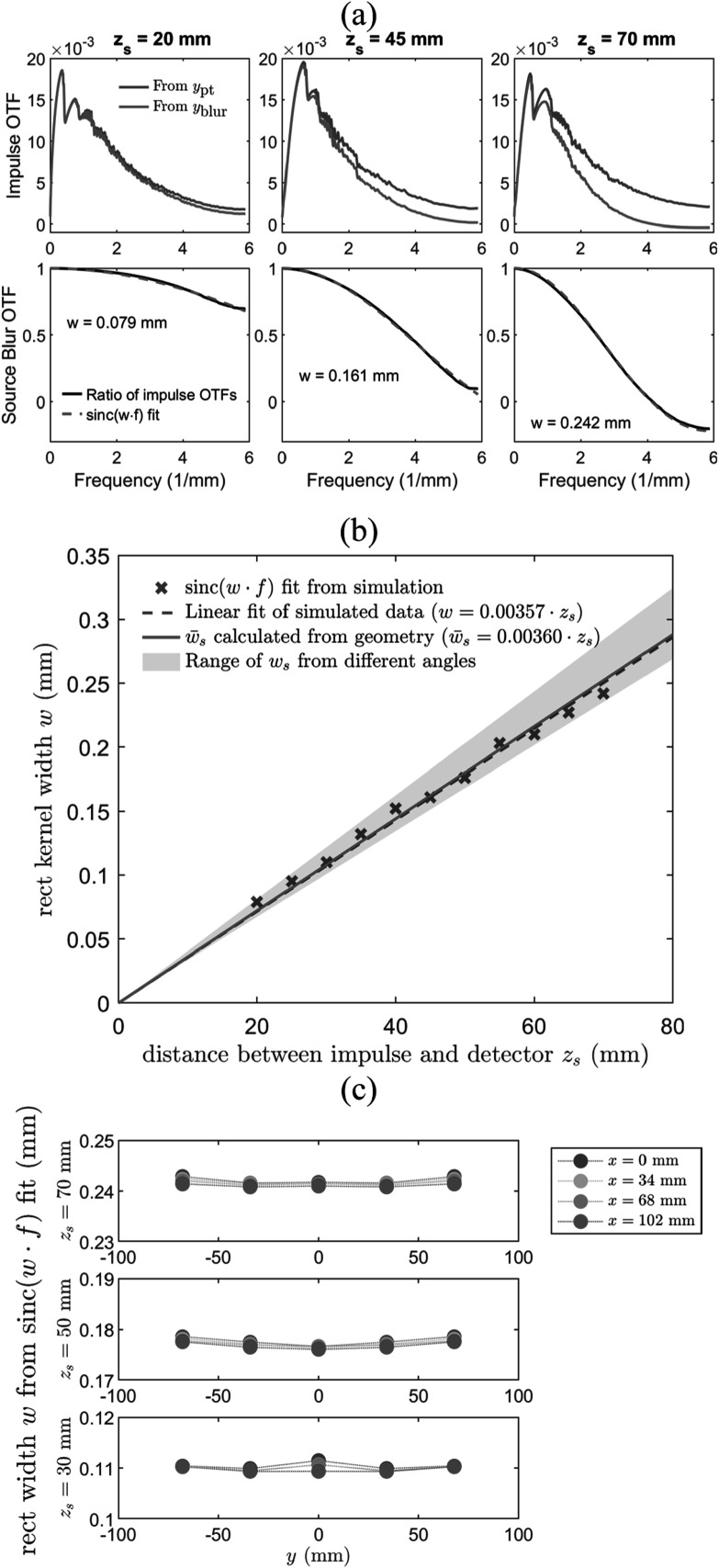
(a) Example impulse OTFs and source blur OTFs for three distances (${z}_{s}$) between the impulse and the detector in the simulation study of blur kernel modeling. (b) The comparison of sinc-fit estimated and analytically calculated blur kernel lengths. The shaded region shows the range of ${w}_{s}$ values, the blur kernel lengths from individual scan angles. (c) The sinc-fit estimated blur kernel lengths for different $x$ and $y$ locations at different ${z}_{s}$.

Figure 5(a) shows the example impulse OTFs and source blur OTFs in the simulation study of the in-plane blur kernel modeling. The impulse was placed close to the chest wall at different heights ${z}_{s}.$ The impulse was centered in $y$-direction, so its PSF and OTF were both real and symmetric. Figure 5(b) shows the scatter plot of the sinc-fit estimated blur kernel length versus ${z}_{s}.$ The data points exhibited a good linear relationship (linear fitting result: $w=0.00357\cdot {z}_{s},$ correlation coefficient = 0.998, *p* < 0.0001). The linear fit of the data points had an almost perfect alignment with the analytically calculated kernel length ${\mathop{w}\limits^{\unicode{x00305}}}_{s}=0.00360\cdot {z}_{s}.$

The shaded region in figure 5(b) shows the range of ${w}_{s},$ the lengths of blur kernels from individual scan angles. For the Siemens Mammomat Inspiration DBT system that acquired 25 PVs from −25° to 25°, ${w}_{s}$ differed from ${\mathop{w}\limits^{\unicode{x00305}}}_{s}$ by −6.7% at 0° (lower bound in figure 5(b)) and 12.7% at 25° (upper bound in figure 5(b)). In other words, the variation of ${w}_{s}$ was small compared to the mean ${\mathop{w}\limits^{\unicode{x00305}}}_{s}.$ We also moved the impulse to different $x,$ $y,$ and $z$ locations in the simulation. As shown in figure 5(c), the sinc-fit kernel sizes were very close with differences less than 1.3% from the mean and were almost shift-invariant for a given ${z}_{s}.$ These observations justified our simplification of source model of ignoring ${w}_{0}^{{\prime\prime} }$ and averaging the kernel lengths over all scan angles, resulting in a shift-invariant 1D line kernel over the area of a reconstructed DBT plane at a given ${z}_{s}.$

### 4.2. DDPM unconditional image generation

**Figure 6.**
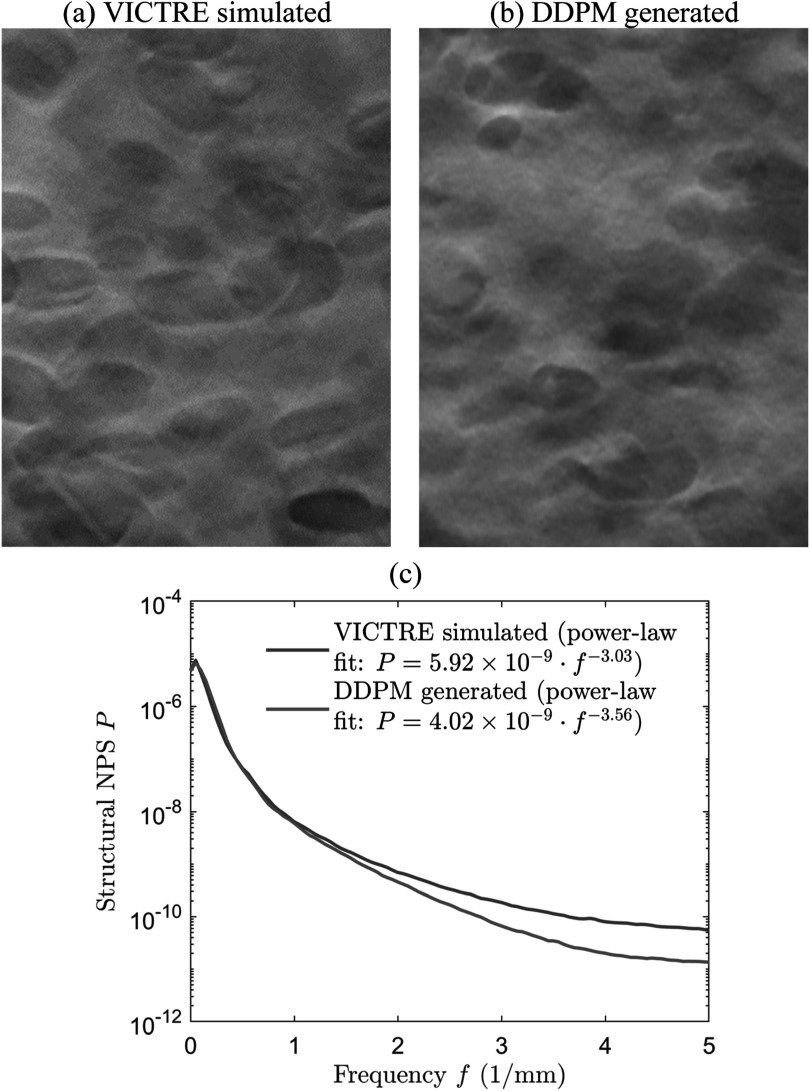
(a) DBT slice from the virtual phantoms used in the DDPM training set. (b) DBT slice generated by unconditional DDPM sampling. Image sizes are 300 × 400 pixels (25.5 mm × 34 mm). (c) Structural NPS of VICTRE simulated and DDPM generated images, averaged over 10 samples for each condition. The power-law fit used the whole frequency range, excluding the zero frequency.

To demonstrate the ability of DDPM to produce high-quality DBT images, we ran unconditional DDPM sampling to draw samples from the prior distribution $p\left({{\boldsymbol{x}}}_{{\mathrm{true}}}\right).$ Figure 6(a) shows an example DBT slice from the DDPM training set. Figure 6(b) shows an example of DDPM generated sample. The DDPM generated images had natural heterogeneous background textures resembling the characteristics of the training images and were free from artifacts. The generated DBT images could be considered to be 1.0 mm slices, similar to the training samples. The structural noise power spectrum (NPS) (Gao *et al* 2021a) of the DDPM generated images exhibited a power-law form as for mammograms (Burgess 1999) and was close to the NPS of the VICTRE simulated training images, as shown in figure 6(c). The exponent values of the power-law fitting to the NPS curves for the VICTRE simulated images and the DDPM generated images were 3.03 and 3.56, respectively.

### 4.3. Image deblurring with generative diffusion

#### 4.3.1. Parameter selection of DDPM posterior sampling

**Figure 7.**
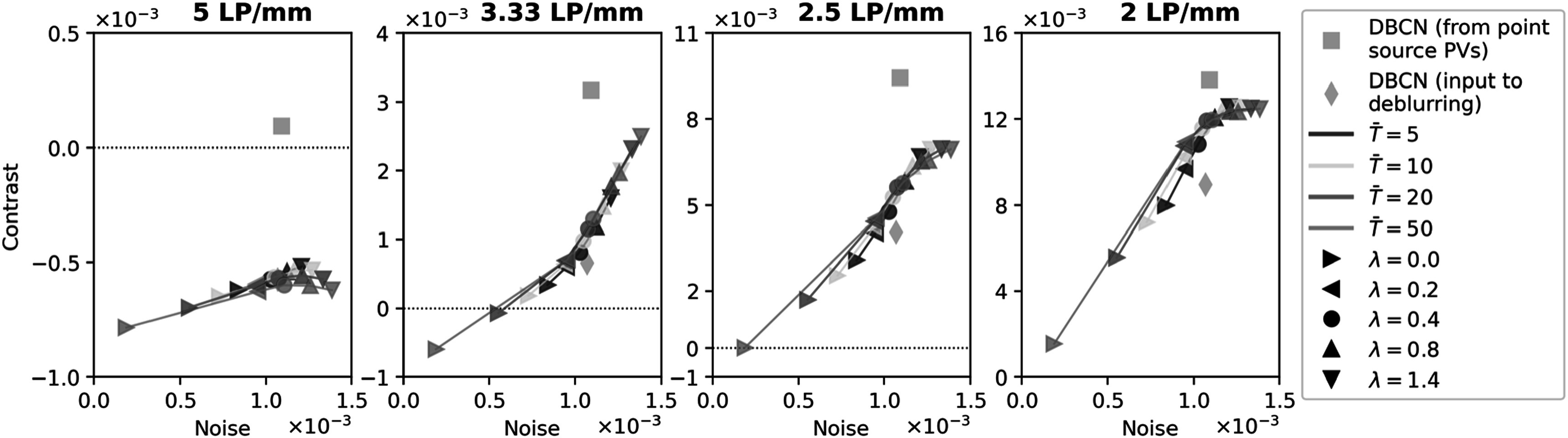
Contrast-vs-noise plots of the LP test object in the 80 mm scattered dense test phantom, showing the dependence on the parameters of the proposed deblurring method with generative diffusion.

To select the number of sampling steps $\mathop{T}\limits^{\unicode{x00305}}$ and the weight parameter $\lambda $ in DDPM posterior sampling, we did a grid search by varying $\mathop{T}\limits^{\unicode{x00305}}$ = 5, 10, 20, 50, and $\lambda $ = 0.0, 0.2, 0.4, 0.8, 1.4. We positioned the LP test object at ${z}_{s}$ = 70 mm in the 80 mm scattered dense test phantom (note the 20 mm gap between the detector and the bottom of the compressed breast) and deblurred its DBCN reconstructed image using these parameter settings. Figure 7 shows the contrast-vs-noise plots of the LPs. The LPs with 5 LP/mm had severe blurring due to their narrow spacing, and the frequency was close to the Nyquist frequency associated with the voxel size (5.88 LP/mm). Deblurring could not recover their resolution, resulting in always negative contrasts. Therefore, we focused our attention on LP frequencies lower than 5 LP/mm.

When $\lambda $ = 0.0, the deblurring method simplified to unconditional DDPM sampling. In this situation, the blur of LPs became more severe as $\mathop{T}\limits^{\unicode{x00305}}$ increased. As we increased $\lambda ,$ the LP contrast improved due to the high frequency boosting from the gradient of the data-fit term. However, this enhancement also amplified background noise. Additionally, the enforcement of data fidelity at each sampling step became stronger, making the impact of $\mathop{T}\limits^{\unicode{x00305}}$ less apparent. To balance between contrast enhancement and noise control, we selected $\lambda $ = 0.4 and $\mathop{T}\limits^{\unicode{x00305}}$ = 20 for the subsequent sections of this study. This parameter setting improved the image resolution after deblurring while maintaining the same image noise level as the blurry input.

#### 4.3.2. Effect of breast densities on deblurring

**Figure 8.**
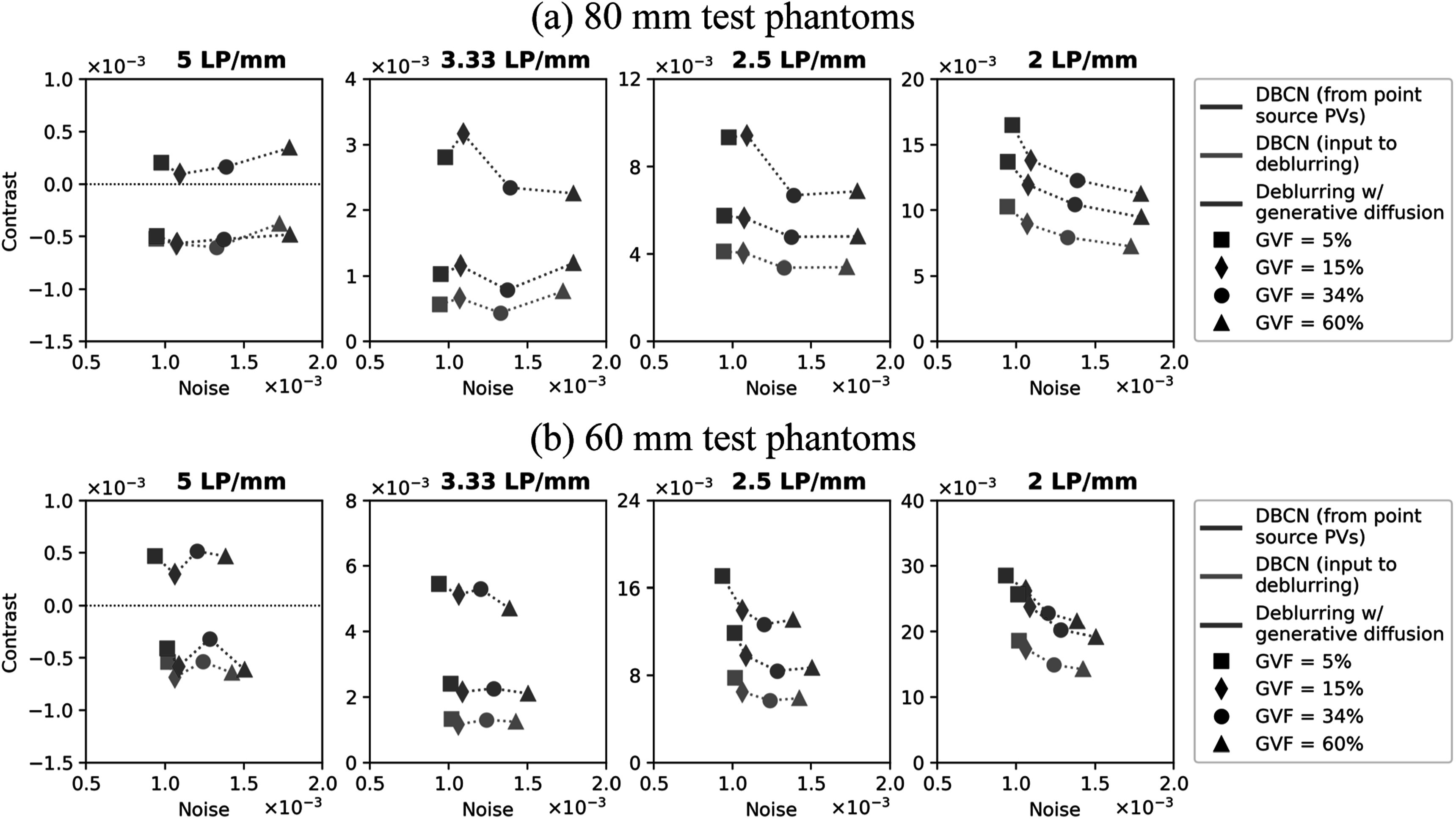
Contrast-vs-noise plots of the LP test object in (a) the 80 mm test phantoms and (b) the 60 mm test phantoms, demonstrating the effect of breast density on the performance of the proposed deblurring method with generative diffusion.

To investigate the effect of breast density on the deblurring performance, we applied the proposed deblurring method to the 8 test phantoms that were 80 mm and 60 mm thick with 4 breast density settings: 5%, 15%, 34%, and 60% GVF. We placed the LP test object at ${z}_{s}$ = 70 mm in these phantoms, which was 30 mm and 10 mm, respectively, deep inside the breasts from the compression paddle. Figure 8 shows the contrast-vs-noise plots of the LPs for the DBCN images and the deblurred images. The LP contrasts in the 80 mm phantoms were generally lower than those in the 60 mm phantoms because more scatter in thicker phantoms and more severe inter-plane artifacts caused a loss of object contrast. For either the 80 mm or the 60 mm phantoms, as GVF increased, the image noise increased due to the AEC mechanism in the MC-GPU simulation. In particular, the same breast thickness used the same mean glandular dose adjusted by the AEC. Hence, dense breasts absorbed more x-rays and had fewer transmitted x-rays, leading to higher image noise. Regardless, the proposed deblurring method with generative diffusion consistently improved the LP contrasts, demonstrating its robustness and flexibility to handle various image noise levels and breast densities. The relative trends of the improvement for the 60 mm and 80 mm phantoms were similar. While we mainly used the scattered dense test phantoms (GVF = 15%) in other sections of this study, our findings affirmed the applicability of the proposed method to a broader range of breast densities.

#### 4.3.3. Effect of test object heights above detector

**Figure 9.**
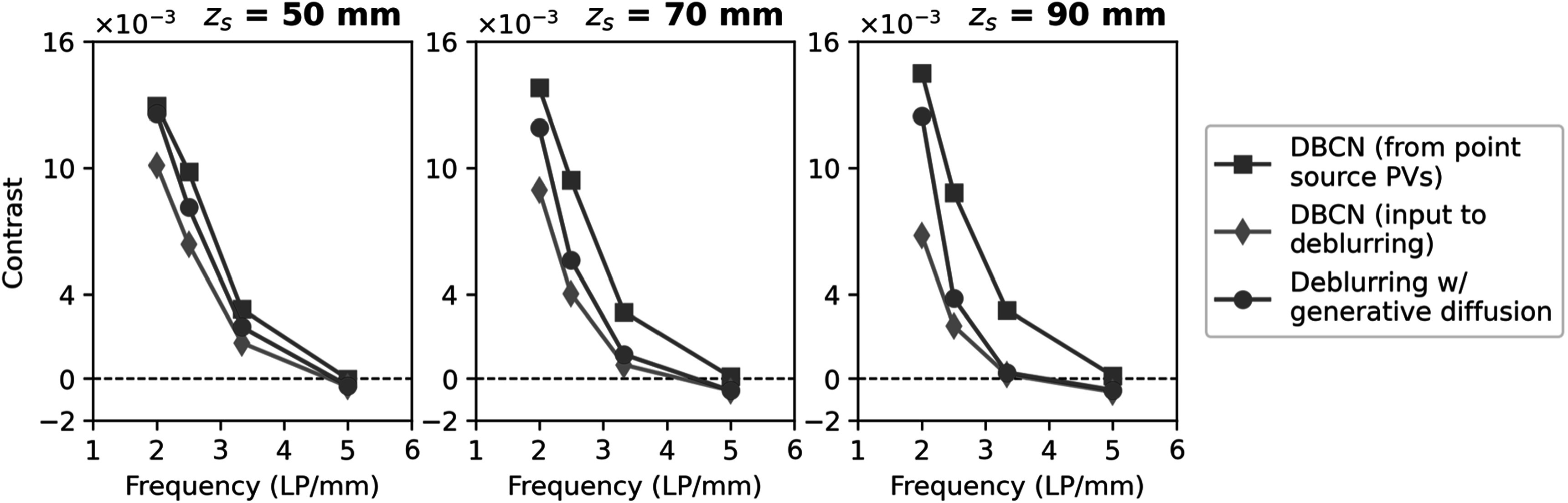
Contrast-vs-frequency plots of the LP test object in the 80 mm scattered dense test phantom, demonstrating the effect of the object height above detector ${z}_{s}$ on the performance of the proposed deblurring method with generative diffusion.

Due to the increased geometric unsharpness, the x-ray source blur increased as the DBT slice became closer to the x-ray source. To assess its impact on the proposed deblurring method, we placed the LP test object at ${z}_{s}$ = 50 mm, 70 mm, and 90 mm in the 80 mm scattered dense test phantom, which was 50 mm, 30 mm, and 10 mm, respectively, deep inside the breast from the compression paddle. The magnification factors of these slices were 1.08, 1.12, and 1.16, respectively. Figure 9 shows the contrasts versus LP frequency for the DBCN images and the deblurred images. These contrast-vs-frequency plots resembled the modulation transfer function (MTF) curves commonly used in assessing radiographic systems, albeit with the signals represented by rectangular LPs instead of sine waves. Also, our LPs were made with calcium oxalate instead of lead in the MC-GPU simulation.

The DBCN images reconstructed from point source PVs served as the reference standard, where the LP contrasts remained almost the same irrespective of ${z}_{s}$ due to the absence of source blur. The DBCN images reconstructed from blurry source PVs had a reduction in LP contrasts compared to the reference standard. This reduction was more pronounced when ${z}_{s}$ increased. The proposed deblurring method with generative diffusion successfully enhanced the LP contrasts and improved the image spatial resolution across all three conditions. Nevertheless, there remained room for improvement with respect to the reference standard, especially for the challenging scenarios where the test object was closer to the x-ray source. The deblurring method was not effective for LPs that were entirely blurred and had nearly zero or negative contrasts.

#### 4.3.4. Comparison of deblurring methods

**Figure 10.**
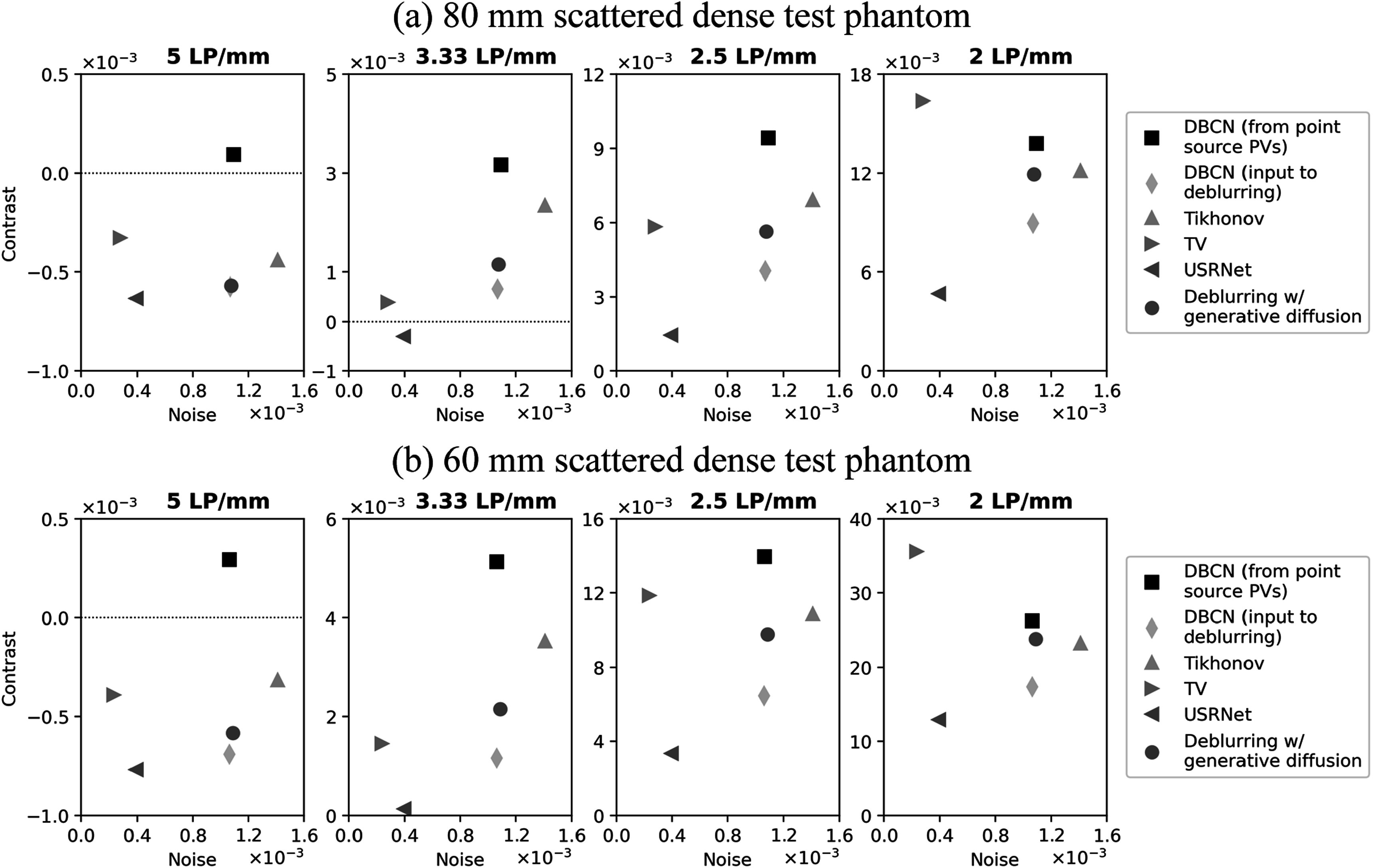
Contrast-vs-noise plots of the LP test object in (a) the 80 mm scattered dense test phantom and (b) the 60 mm scattered dense test phantom, for comparing the deblurring performance of different methods on the DBCN-reconstructed images.

**Figure 11.**
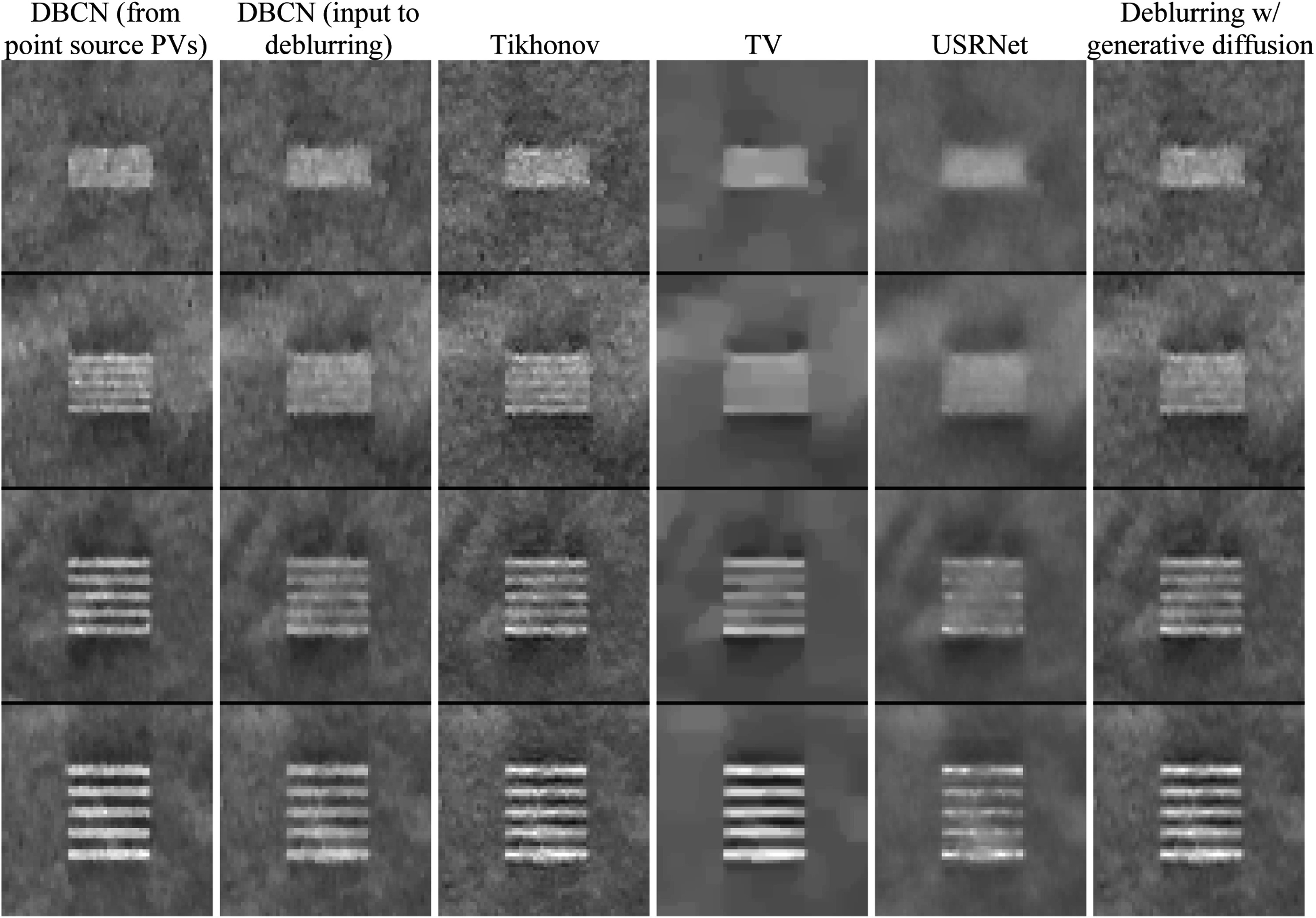
Example ROIs of the LP test object in the 80 mm scattered dense test phantom for comparing different deblurring methods on the DBCN-reconstructed images. From top to bottom, the LPs have spatial frequencies of 5 LP/mm, 3.33 LP/mm, 2.5 LP/mm, and 2 LP/mm. The ROI sizes are 60 × 60 pixels (5.1 mm × 5.1 mm).

We evaluated the performance of different deblurring methods on the DBCN-reconstructed images using the 80 mm and the 60 mm scattered dense test phantoms with the LP test object placed at ${z}_{s}$ = 70 mm. Figure 10 shows the contrast-vs-noise plots. Figure 11 displays the example LP ROIs from the 80 mm phantom.

Comparing the results from the 80 mm and the 60 mm phantoms, the overall trends were similar, as observed in section 4.3.2. Tikhonov regularized deblurring involved an inverse filter that inevitably enhanced the LP contrast and image noise at the same time. We tried a range of ${\mu }_{{\mathrm{Tik}}}$ values and set it to 0.05. TV regularization (${\mu }_{{\mathrm{TV}}}$ = 0.0005) performed very poorly on breast images, mainly because TV caused piecewise-constant patchy artifacts and could not characterize the ill-defined boundaries of soft tissue. While USRNet effectively smoothed the images, it failed to preserve the LP signals in the images. The proposed deblurring method with generative diffusion achieved an improvement in the LP contrast while maintaining a similar image noise level as the DBCN images. According to our visual judgment, it also retained the natural appearance of the tissue background without introducing artifacts.

## 5. Discussion

As mentioned in section 2.2, it is challenging to address source motion blur by directly using the modified system matrix ${\boldsymbol{AB}}$ for forward and backward projections given the limited-angle and underdetermined nature of the DBT reconstruction problem. We conducted a simulation study to demonstrate the limitations of that approach. The simulation setup was the same as section 3.2, where we created an impulse object and generated noise-free ${{\boldsymbol{y}}}_{{\mathrm{pt}}}$ and ${{\boldsymbol{y}}}_{{\mathrm{blur}}}$ PVs. The impulse was placed close to the chest wall and centered in $y$ at ${z}_{s}$ = 70 mm. We reconstructed the impulse using gradient descent for the following formulations: (1) $\mathop{{\mathrm{argmin}}}\limits_{{\boldsymbol{v}}}\frac{1}{2}{\unicode{x02016}{\boldsymbol{Av}}-{{\boldsymbol{y}}}_{{\mathrm{pt}}}\unicode{x02016}}^{2},$ (2) $\mathop{{\mathrm{argmin}}}\limits_{{\boldsymbol{v}}}\frac{1}{2}{\unicode{x02016}{\boldsymbol{Av}}-{{\boldsymbol{y}}}_{{\mathrm{blur}}}\unicode{x02016}}^{2},$ (3) $\mathop{{\mathrm{argmin}}}\limits_{{\boldsymbol{v}}}\frac{1}{2}{\unicode{x02016}{\boldsymbol{ABv}}-{{\boldsymbol{y}}}_{{\mathrm{blur}}}\unicode{x02016}}^{2}.$ We also investigated a fourth condition for the idea of introducing sub-focal spots within the blurry source: (4) $\mathop{{\mathrm{argmin}}}\limits_{{\boldsymbol{v}}}\frac{1}{2}{\unicode{x02016}{{\boldsymbol{A}}}_{{\mathrm{sub}}}{\boldsymbol{v}}-{{\boldsymbol{y}}}_{{\mathrm{blur}}}\unicode{x02016}}^{2}.$ This idea was similar to the use of ${\boldsymbol{AB}}$ and was shown to be effective for source blur modeling in CT (Fu *et al* 2013, Majee *et al* 2022). The modified system matrix ${{\boldsymbol{A}}}_{{\mathrm{sub}}}$ was defined as

**Figure 12.**
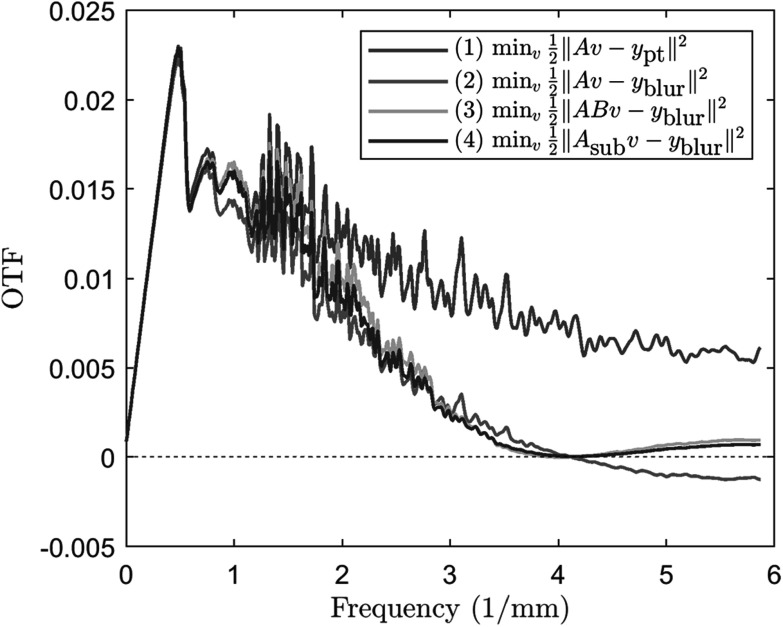
The impulse OTFs that demonstrate the limitations of using ${\boldsymbol{AB}}$ or ${\boldsymbol{A}}$_sub_ to model source motion blur for DBT reconstruction.

\begin{eqnarray*}{{\boldsymbol{A}}}_{{\mathrm{sub}}}=\frac{1}{{N}_{{\mathrm{sub}}}}\sum _{{i}_{{\mathrm{sub}}}=1}^{{N}_{{\mathrm{sub}}}}{\boldsymbol{A}}\left({\delta }_{{i}_{{\mathrm{sub}}}}\right),\end{eqnarray*}

where ${N}_{{\mathrm{sub}}}$ is the number of sub-focal spots, ${\boldsymbol{A}}\left(\delta \right)$ is the perturbed system matrix of ${\boldsymbol{A}}$ obtained by offsetting the scan angles by $\delta ,$ and ${\delta }_{{i}_{{\mathrm{sub}}}}=\frac{{w}_{0}}{{D}_{{SO}}}\frac{1}{{N}_{{\mathrm{sub}}}}\left({i}_{{\mathrm{sub}}}-\frac{{N}_{{\mathrm{sub}}}+1}{2}\right)$ in radians for ${i}_{{\mathrm{sub}}}=1,\,\ldots ,\,{N}_{{\mathrm{sub}}}.$ Figure 12 shows the OTFs of the extracted impulse PSF profiles in the $y$-direction. Although condition (1) was from the point source PV using the point source projector, it still largely deviated from the ideal OTF (a horizontal line with a value of one) with large oscillations even in the absence of noise. The decrease in OTF from condition (1) to condition (2) was due to the source motion blur. Their ratio corresponded to the smooth source blur OTF that was fitted by sinc functions in section 4.1. Condition (3) and (4) demonstrated that both ${\boldsymbol{AB}}$ and ${{\boldsymbol{A}}}_{{\mathrm{sub}}}$ were able to correct the negative phases at high frequency bands caused by the blur. However, the OTF magnitudes were not significantly improved and remained considerably lower than condition (1). This result suggested that directly using ${\boldsymbol{AB}}$ or ${{\boldsymbol{A}}}_{{\mathrm{sub}}}$ for (unregularized) reconstruction cannot recover the loss in resolution caused by source motion blur in DBT.

The DDPM network uses an unsupervised training approach that solely requires high-quality images. To apply DDPM for deblurring, we simply need to integrate the gradient of the data-fit term into the DDPM sampling process, requiring no re-training or fine-tuning of the DDPM network. This unique feature endows DDPM much flexibility in terms of training data preparation because there is no need for paired low-quality and high-quality images. Moreover, it also makes the DDPM regularization very robust in that a single trained network can be applied to not only deblurring, but also other image restoration tasks as long as the specific task can be defined by a degradation operator like the blur matrix ${\boldsymbol{B}}$ in (7).

Although the simulated training images do not contain LPs, the proposed deblurring method with the trained diffusion network is able to preserve the LP test objects and enhance their contrasts. This advantage is crucial, especially considering that MC signals in DBT images are sparse and small so they may be difficult for a network to learn. Deblurring by DDPM posterior sampling may help preserve the signals of interest such as MCs when a discrepancy exists between the training data and test data. The use of multiple diffusion steps in each deblurring process also ensures more gradual alterations of image content. In contrast, the end-to-end trained USRNet processes the images in a single step, resulting in an abrupt change in image content and a failure to retain the LPs.

To account for the possible alignments of LPs with the detector pixel array, we created shifted LPs in the test phantom. Another idea is to create LPs with an orientation slightly tilted with respect to the detector pixels and produce an oversampling of LPs. This idea proves to be challenging under the current settings because the virtual phantoms are defined on discrete voxels instead of continuous space and it is difficult to create smooth tilted LPs in the phantoms given the finite voxel resolution. In our preliminary study, we also investigated other designs of test objects, such as closely spaced bead pairs mimicking MCs. Compared with LPs, bead pairs were less discernable in the images and their quantitative metrics suffered from large variations due to the small sizes and noise. Therefore, we did not use bead pairs as our test objects.

We made a compromise by deblurring the reconstructed images and achieved moderate improvement in image sharpness. Post-processing deblurring has the advantage that it is applicable to DBT obtained from any reconstruction techniques. Nonetheless, post-processing deblurring may not be the optimal solution because the measured PVs are not exploited in the deblurring step. Future research is required to further improve the image resolution, especially for the image slices closer to the x-ray source where the blur is more severe.

We trained and tested the deblurring method using VICTRE simulated images. Besides the realism of the VICTRE phantoms, one of the main advantages of using simulation data is the availability of DBT scans from an ideal x-ray source. The point source DBT images can be used as ground truth, which are otherwise impossible to obtain from real scans, for either network training or algorithm evaluation. Our deblurring method has not been tested with real patient images due to the unavailability of data. Future work should apply this method to real patient DBT images with x-ray source motion blur to evaluate its effectiveness in clinical scenarios. We also acknowledge the importance of evaluating the proposed deblurring method using commercial phantoms with typical objects that simulate MCs. However, we do not have access to real data acquired with a continuous-motion DBT system at this time, and the use of commercial phantoms is not feasible within the scope of our current investigation. We will consider this aspect as part of our future work.

## 6. Conclusion

In this study, we introduced a new approach for modeling x-ray source motion blur in DBT imaging. We derived the in-plane source blur kernel for the reconstructed DBT slices based on imaging geometry and showed that it could be approximated by a shift-invariant kernel over the DBT plane at a given depth. We conducted a simulation study to validate its accuracy. Our simulation also underscored the limitations of modifying the system matrix to model source blur in DBT reconstruction, whether by incorporating the source blur matrix or introducing subsampling focal spots. In view of these limitations, we proposed a post-processing deblurring method with generative diffusion for the reconstructed DBT images using the known blur kernel. The quantitative results demonstrated that our deblurring method improved spatial resolution while maintaining the same level of image noise when applied to DBT images reconstructed with detector blur and correlated noise modeling. Future research can explore further refinements of the deblurring technique and investigate its application to human subject data for improving the diagnostic accuracy of DBT imaging.

## Data Availability

The data cannot be made publicly available upon publication because the cost of preparing, depositing and hosting the data would be prohibitive within the terms of this research project. The data that support the findings of this study are available upon reasonable request from the authors.
